# A physiologically based pharmacokinetic model for V937 oncolytic virus in mice

**DOI:** 10.3389/fphar.2023.1211452

**Published:** 2023-09-13

**Authors:** Sara Peribañez-Dominguez, Zinnia P. Parra-Guillen, Tomoko Freshwater, Iñaki F. Troconiz

**Affiliations:** ^1^ Department of Pharmaceutical Technology and Chemistry, School of Pharmacy and Nutrition, University of Navarra, Pamplona, Spain; ^2^ Navarra Institute for Health Research (IdiSNA), Pamplona, Spain; ^3^ Quantitative Pharmacology and Pharmacometrics Immune/Oncology (QP2-I/O) Merck & Co., Inc., Rahway, NJ, United States; ^4^ Institute of Data Science and Artificial Intelligence (DATAI), University of Navarra, Pamplona, Spain

**Keywords:** PBPK, oncolytic virus, physiologically based, viral kinetics, mechanistic modeling, biodistribution

## Abstract

**Introduction:** Oncolytic viruses (OVs) represent a novel therapeutic strategy in oncology due to their capability to selectively infect and replicate in cancer cells, triggering a direct and/or immune-induced tumor lysis. However, the mechanisms governing OV pharmacokinetics are still poorly understood. This work aims to develop a physiologically based pharmacokinetic model of the novel OV, V937, in non-tumor-bearing mice to get a quantitative understanding of its elimination and tissue uptake processes.

**Materials and methods:** Model development was performed using data obtained from 60 mice. Viral levels were quantified from eight tissues after a single intravenous V937 dose. An external dataset was used for model validation. This test set included multiple-dose experiments with different routes of administration. V937 distribution in each organ was described using a physiological structure based on mouse-specific organ blood flows and volumes. Analyses were performed using the non-linear mixed-effects approach with NONMEM 7.4.

**Results:** Viral levels showed a drop from 10^8^ to 10^5^ copies/µg RNA at day 1 in blood, reflected in a high estimate of total clearance (18.2 mL/h). A well-stirred model provided an adequate description for all organs except the muscle and heart, where a saturable uptake process improved data description. The highest numbers of viral copies were observed in the brain, lymph node, kidney, liver, lung, and spleen on the first day after injection. On the other hand, the maximum amount of viral copies in the heart, muscle, and pancreas occurred 3 days after administration.

**Conclusion:** To the best of our knowledge, this is the first physiologically based pharmacokinetic model developed to characterize OV biodistribution, representing a relevant source of quantitative knowledge regarding the *in vivo* behavior of OVs. This model can be further expanded by adding a tumor compartment, where OVs could replicate.

## 1 Introduction

Oncolytic viruses (OVs) possess the ability to selectively infect cancer cells, inducing their death (Haseley et al., 2009; Goldufsky et al., 2013), thus avoiding the destruction of non-neoplastic tissue. Some viruses have natural selectivity for tumor cells, while others need to be genetically modified in order to detect overexpressed receptors on tumor cells or be dependent on tumor transcription or signaling pathways. Once a tumor cell is infected, oncolytic viruses use cellular translation and transcription elements to replicate (Titze et al., 2017). Subsequently, OVs induce cell death by direct virus-mediated cytotoxicity, through immune-mediated mechanisms, or through indirect routes, such as the destruction of tumor blood vessels or specific activities carried out by genetically encoded proteins. Therefore, oncolytic viruses can, in principle, offer selective tumor targeting, high tumor exposure due to viral replication, and immune activation to combat tumor evasion (Parato et al., 2005; Russell and Peng, 2007; Titze et al., 2017).

The approval of Oncorine in China for the treatment of neck cancer (2005) and the FDA approval of T-VEC for metastatic melanoma (2015) (Garber, 2006; Greig, 2016) have been important milestones in the development of these types of therapies. As a result, there has been an increase in research conducted in this field over the past decades. Currently, more than 10 clinical trials of OVs are active in advanced stages of development (phases II and III) (McCarthy et al., 2019).

V937 is a positive single-stranded RNA virus enclosed in an icosahedral, 30 nm in diameter. It is an unmodified bioselected strain of Coxsackievirus A21, an enterovirus of the Picornaviridae family responsible for common cold in humans, which has shown oncolytic activity against solid tumors. Its efficacy has been demonstrated both *in vitro* and preclinically in several types of cancer, such as melanoma, multiple myeloma, and lung or prostate cancer types (Shafren et al., 2004; Au et al., 2005; 2007; Berry et al., 2008; Skelding et al., 2012). It has been demonstrated that V937 is able to infect cells by interacting with intracellular adhesion molecule 1 (ICAM-1) (Annels et al., 2018). The VP1 capsid protein from V937 contains a hydrophobic canyon that binds to viral entry receptors present on host cells. This receptor is also involved in virus internalization, triggering a conformational change that prompts the release of viral RNA into the host cell. Subsequently, its presence is necessary for the entry of V937 into the cell. Tumor cells present an overexpression of ICAM-1, which provides selectivity to the virus for damaged cells, avoiding the infection of healthy cells (Bradley et al., 2014; Arif, 2018).

The complexity of the different processes that play a relevant role in the anti-tumor response of OVs makes the mechanistic quantitative approach a key element in the development programs of these therapeutics. Several theoretical studies have been conducted on viral dynamics, whereas pharmacokinetics has received very little attention (Karev et al., 2006; Bradley et al., 2014; Parra-Guillen et al., 2021).

Intratumor (IT) administration is the most common route for this type of therapy since it maximizes exposure of the virus to the tumor. However, it has some limitations, such as access to deep lesions such as glioblastoma (Li et al., 2020). On the other hand, intravenous (IV) administration represents an interesting alternative, but limited clinical efficacy has been observed so far using this route. Characterizing the distribution of OVs to the tumor space is crucial to understand not only its *in vivo* response but also its capability to access other organs of the body, which will help to predict their systemic disposition, including catabolism, and increase the understanding of the physiological factors governing OV pharmacokinetics.

Based on the abovementioned considerations, the aim of this work was to develop a preclinical physiologically based pharmacokinetic (PBPK) model for a novel oncolytic virus, leveraging levels of V937 obtained in different organs and literature data on ICAM receptor expression levels. To the best of our knowledge, this work represents the first attempt to develop such types of mechanistic platforms aimed at describing OV biodistribution. Moreover, this platform is not necessarily restricted to OV and could be used for other virus-based therapies.

## 2 Materials and methods

### 2.1 Animals

Data from 60 Hu/Mu ICAM-1 transgenic mice (*n* = 30 male) were used to build the PBPK model (training group), which was externally validated using additional data (validation group) from 99 Hu/Mu ICAM-1 transgenic mice (*n* = 50 male).

The training group included 12–14-week-old mice. The animals were housed with 3–5 mice per cage (an HEPA-filtered Techniplast cage (1145 IVC) connected to a Techniplast Slim Line air handling system) in a PC2 laboratory with a 12/12 light/dark cycle. Bedding made from corn cob (Shepherd’s cob) was used to line the cages (Shepherd Specialty Papers Inc., TN, United States). The mice were fed *ad libitum* with rat and mouse cubes/pellets manufactured by Specialty Feeds, WA, Australia. This standard mouse feed is formulated to be low in fat content (approximately 5%) and is meat free. The airflow in the room was 12–15 air changes per hour, but the airflow in the Techniplast cages was at a rate of 70 changes per hour. The mice were identified by tail marking with a permanent pen.

For the rest of the experiments, the same animal care conditions were reproduced. The age of the mice in the case of the single-dose IV administration experiment was 6–8 weeks. The age of mice in the case of multiple-dose subcutaneous experiments was 8–10 weeks. All animals were euthanized by CO_2_ asphyxiation following tissue and organ extraction.

The study was performed according to an Animal Use Protocol approved by the University of Newcastle Animal Care and Ethics Committee, project license number A2010-143.

### 2.2 Design of experiments

The mice in the training group received a single rapid IV injection containing 2.5 × 10^7^ TCID_50_ (half-maximal tissue culture infectious dose) of V937 through the tail vein. A blood sample was obtained 45 min after administration. Then, 12 animals per day were euthanized at days 1, 3, 7, 10, and 14, and viral levels (copies/µg RNA) were quantified in the following tissues: blood, lung, brain, heart, muscle, pancreas, spleen, liver, kidney, and lymph node.

Data used for model validation were obtained from three independent experiments where multiple doses of V937 were given either intravenously (one experiment) or subcutaneously (two experiments).

In the IV experiment, 1 × 10^8^ TCID_50_ of V937 were injected in the tail vein at days 1, 3, 6, 8, 10, 13, 15, 17, 19, and 22. A blood sample was obtained 45 min after administration. Then, eight animals per day were euthanized at days 6, 24, and 36 after the start of the treatment. Viral levels (copies/µg RNA) were quantified in the following tissues: blood, heart, lung, brain, spleen, kidney, liver, muscle, and pancreas.

In the first subcutaneous experiment, 2.5 × 10^7^ TCID_50_ of V937 were injected alternating between the intercapsular region (between the shoulder blades) and the flanks at days 1, 3, 5, 10, 12, 15, 17, 19, and 22. Blood samples were obtained 30 min after injection and at days 9 and 24. Tissue samples were obtained only at the end of the experiment on day 24 (*n* = 8 mice). Viral levels (copies/µg RNA) were quantified in the following tissues: the heart, lung, brain, spleen, kidney, liver, ileum, gastrocnemius muscle, stomach, lower intestines, and gonads. Similarly, in the second subcutaneous experiment, the same dose level of V937 was given at days 1, 3, 6, 8, 10, 13, 15, 17, 19, and 22. Tissue samples were obtained at days 6 (*n* = 7 mice) and 24 (*n* = 8 mice) from the following organs: the blood, heart, lung, brain, spleen, kidney, liver, muscle, pancreas, gonads, ileum, stomach, and lymph.

**FIGURE 1:**
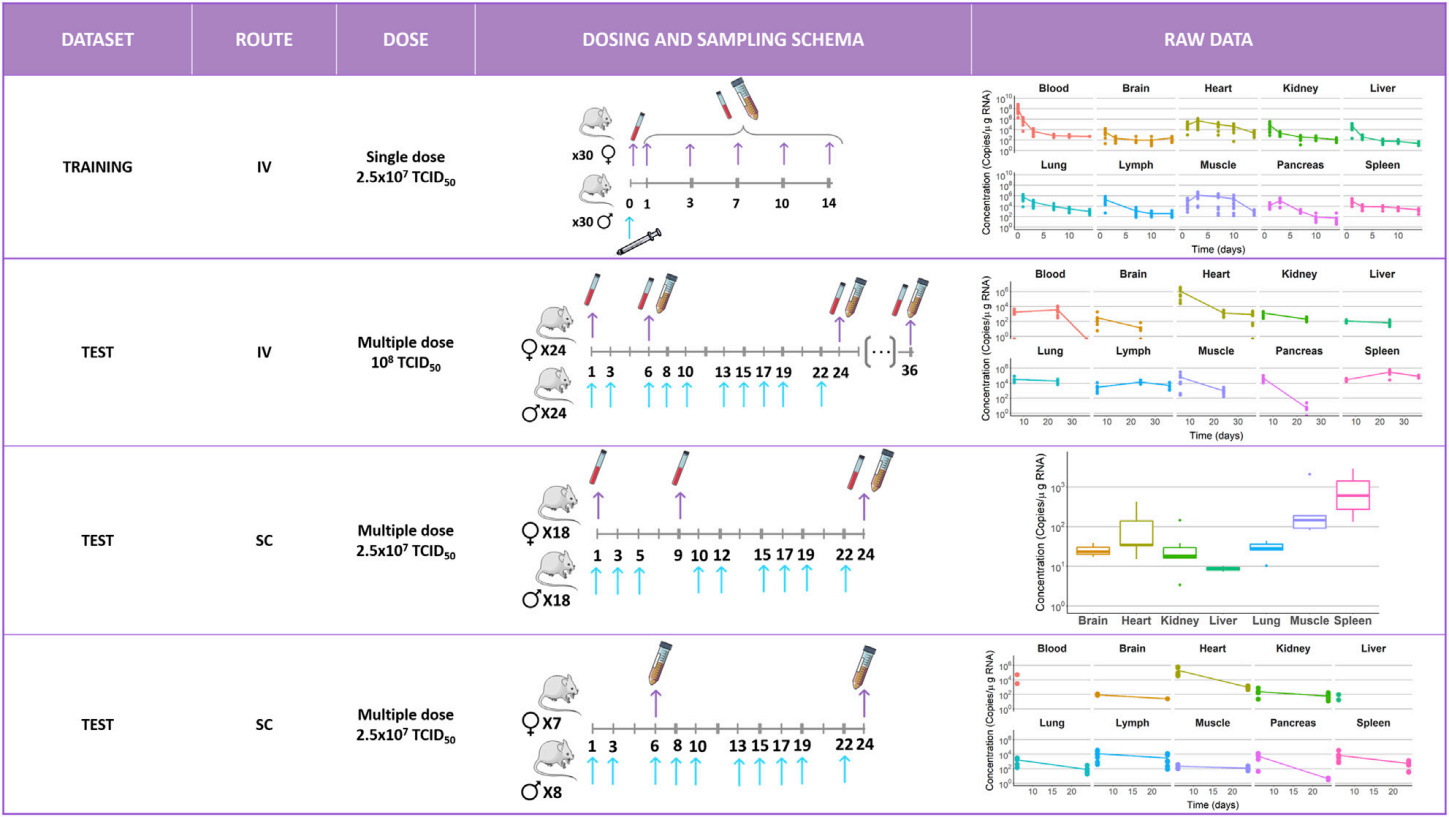
Schematic representation of the different experimental setups included in the current evaluation along with the corresponding longitudinal raw data profiles measured in different tissues. Blue arrows indicate administration times. Purple arrows indicate sample extraction times. Each color corresponds to an organ as follows: 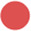
= blood, 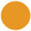
= brain, 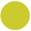
= heart, 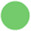
= kidney, 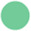
= liver, 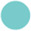
= lung, 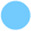
= lymph, 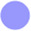
= muscle, 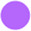
= pancreas, and 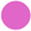
= spleen. IV, intravenous; SC, subcutaneous; and TCID_50_ = half-maximal tissue culture infectious dose.

Figure 1 provides a schematic representation of the different experimental setups included in the current evaluation along with the corresponding longitudinal raw data profiles.

A transformation of units of the administered dose to copies was performed using the conversion factor estimated by Parra-Guillen et al. to maintain coherence between administration and observation units (TCID_50_ and copies/µg RNA, respectively). This study had access to measurements of V937 in serum over time in both units: copies/mL and TCID_50_/mL. A good correlation was observed between these two measurement types, leading to the conversion factor of 170 copies/TCID_50_.

### 2.3 Analytical determination

V937 tissue concentrations were determined using a quantitative reverse transcriptase–polymerase chain reaction (qRT-PCR) method. The limit of detection was 5.3 × 10^2^ copies/µg RNA for all experiments except for the quantification of blood samples obtained in the last experiment, which was 1.5 × 10^3^ copies/mL.

### 2.4 Data analysis

The non-linear mixed-effects modeling approach using the software NONMEM 7.4 with the Laplacian estimation method and interaction was used for data analysis. Observations below the limit of detection were analyzed as censored information, maximizing the likelihood of an observation being below the limit of quantification (M3 method (Beal, 2001)). Inter-animal variability in the drug-specific parameters (see the following) was modeled exponentially. The residual error was modeled with an additive model in the logarithmic scale, as a logarithmic transformation of the data was performed during the analysis.

#### 2.4.1 Model selection

Selection between competing models was carried out considering several indicators: i) the minimum value of the objective function value (OFV), which approximates to 2x log-likelihood and where reductions in 3.84 and 6.61 points in the OFV between two nested models are associated with model improvement at the 5% and 1% levels of statistical significance, respectively, and ii) goodness-of-fit (GOF) plots, which allow to visually judge, among others aspects, the agreement between model predictions and observations.

#### 2.4.2 Model evaluation

The ability of the selected model to describe the typical data profiles and their dispersion in the animals from the training dataset was explored by simulating 1,000 studies with the same design characteristics as the original one. For each simulated dataset, the 5^th^, 50^th^, and 95^th^ percentiles of the simulated concentrations were calculated at each sampling time and for each tissue. Then, the median of the aforementioned percentiles was plotted together with the corresponding percentiles calculated from the raw data and area covering the 95% prediction intervals of the 50^th^ percentile. Precision of model parameter estimates was evaluated using the log-likelihood profile method (Sheiner, 1986). A sensitivity analysis was performed to evaluate the impact on AUC_0-tend_ for each tissue varying model parameters ±25% of the point estimates.

#### 2.4.3 Model validation

The model was validated using data from the aforementioned validation groups. The typical profiles generated using the selected model structure and the corresponding parameter estimates following the experimental conditions used in the validation groups were visually contrasted with the raw data.

### 2.5 Physiologically based pharmacokinetic models

**FIGURE 2:**
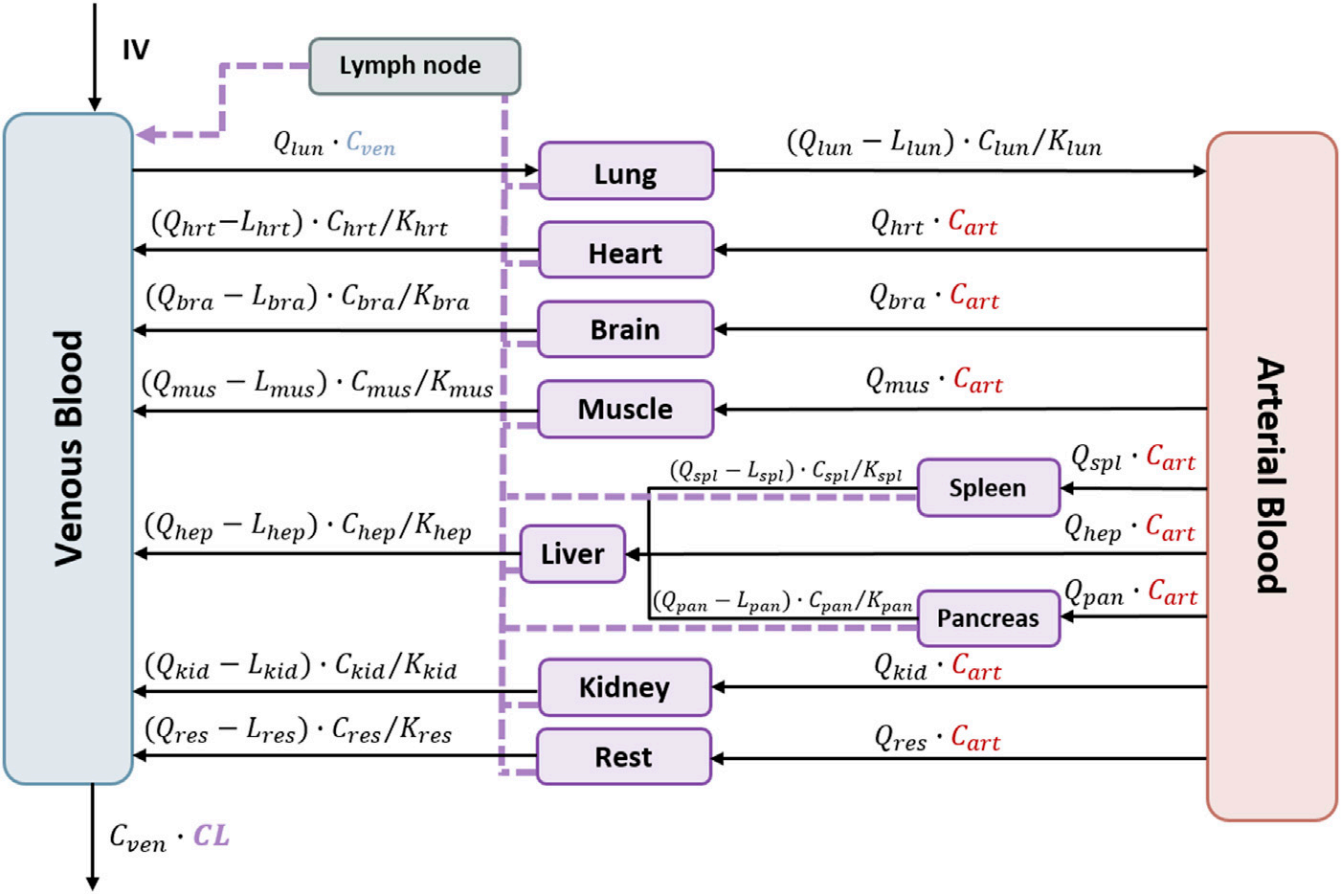
Schematic representation of the perfusion-limited distribution of the physiologically based pharmacokinetic model for V937. The model represents all organs connected by venous and arterial blood. Lymphatic flow is represented by the dashed line. Intravenous administration is represented as IV. Q_org_, organ flow rates; C_org_, organ viral concentrations; L_org_, lymph flow rates; KP_org_, partitions coefficients; CL, total clearance. Each organ corresponds to the following abbreviations: veins = ven, arteries = art, lung = lun, heart = hrt, brain = bra, muscle = mus, spleen = spl, pancreas = pan, liver = hep, kidney = kid, and rest = res.

Figure 2 schematically represents the structure of the physiologically based pharmacokinetic model that was initially fitted to the data. In that model, perfusion-limited distribution is assumed for all organs. The following general Eq. 1 characterizes the rate of change of the viral load in each organ. Supplementary Appendix (Supplementary Data Sheet S1) lists the full set of model equations, while the main NM-TRAN code has been included in Supplementary Data Sheet S3.

$$\frac{{dA}_{org}}{dt}=Q_{org}∙\frac{A_{blood}}{V_{blood}}-\left(Q_{org}-L_{org}\right)∙\frac{A_{org}}{V_{org}}/{KP}_{org}-L_{org}∙\frac{A_{org}}{V_{org}}.$$ (1)

Here, *A*_*org*_ is the viral load (copies) in that specific organ, *Q*_*org*_ and *L*_*org*_ represent the blood and lymph flows, respectively, *V*_*org*_ corresponds to the volume, and *KP*_*org*_ is the partition coefficient of the lung, brain, heart, muscle, pancreas, spleen, liver, or kidney. Values of blood and organ volumes were calculated according to the weight of the mice using the algorithms implemented in PK-sim^®^ (Open Systems Pharmacology Suite 10). Lymph flows were assumed 500 times lower than the corresponding organ blood flow (Shah and Betts, 2012). An additional compartment (rest) merging the remaining organs from the body was included in the model structure. Values of the volume and flow rate corresponding to the rest compartment were calculated from the difference between total body volume and cardiac output and the corresponding values resulting from the sum of all organs from which measurements were available.

Concentrations (copies/mL) were calculated by dividing the amounts in the organ by the respective physiological volume of each organ. As the viral levels were measured in copies/µg RNA, a scaling factor was estimated during the model-building process to maintain consistency between units. These scaling factors represent the µg of RNA per mL of tissue ( $\left.{RNA}_{org,i}\right)$ . Supplementary Table S1 lists the values of blood and lymph flows and organ volumes used in the current investigation.

During model building, the partition coefficients, the scaling factors, and the total clearance (CL) assumed to occur in the systemic circulation (Russell and Peng, 2007; Tan et al., 2017; Parra-Guillen et al., 2021) were the parameters estimated.

The process of virus entry into organs is described by a flow rate. In the case of organs in which a saturation of this flow into the compartment is perceived, flow-limited models were initially used, showing a clear over-prediction of the levels reached. Accordingly, a saturation mechanism was assumed, attributing it to the exceeding of the binding capacity of the ICAM-1, as this receptor is accountable for the distribution of V937 into tissues. That non-linear mechanism was also considered during model development and was implemented as described in the following equation:

$$\frac{{dA}_{org}}{dt}=\frac{{VMAX}_{org,i}}{{Km}_{org,i}+\frac{A_{blood}}{V_{blood}}}∙\frac{A_{blood}}{V_{blood}}-\left(Q_{org,i}-L_{org,i}\right)∙\frac{A_{org,i}}{V_{org,i}}/{KP}_{org,i}-L_{org,i}∙\frac{A_{org,i}}{V_{org,i}},$$ (2)

where *V*_*MAX*_ represents the maximum zero-order input rate constant and *K*_*m*_, the concentration of copies/mL at which 50% of *V*_*MAX*_ is reached. *K*_*m*_ is derived from the ratio between *VMAX* and the organ-specific blood flow rate.

In the case of the subcutaneous administration experiments and according to experimental evidence (Davis and Bugelski, 1998), the lymph was considered as the compartment from which absorption takes place. Characterization of the absorption process took place during model validation.

### 2.6 Software

Dataset organization, graphical exploration of raw data, and model evaluation were performed using R version 4.0.5 through RStudio interface version 1.4.1106. The software NONMEM 7.4 was used for the analysis. Model management was carried out using Pirana 3.0.0. The Xpose R package was used to display VPCs performed using PsN 5.3.0. Modeling software PK-Sim^®^ (Open Systems Pharmacology Suite 10) was used to obtain physiological parameters.

## 3 Results

### 3.1 Data

**FIGURE 3:**
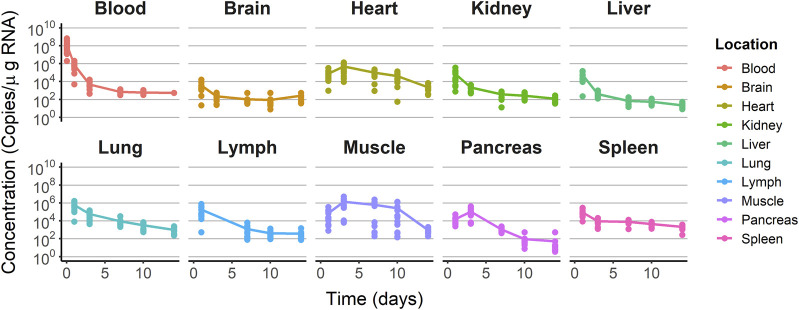
Training dataset concentration–time profiles. Dots represent the observations. Solid lines represent the median tendency in each organ.

The raw longitudinal viral levels vs. time profiles after receiving a single IV dose of V937 were used to establish the model, as presented in Figure 3. After injection, viral levels in blood decreased from 10^8^ copies/µg RNA measured at day 1 to 10^5^ copies/µg RNA measured at day 2. Maximum viral copies were seen already at day 1 after injection in the brain, lymph node, kidney, liver, lung, and spleen. The heart, muscle, and pancreas showed the peak of viral copies at day 3 after administration. With the exception of blood, maximum viral levels ranged from 1.37 × 10^6^ (muscle) to 3.70 × 10^3^ (brain) copies/µg RNA. The last sample was taken 2 weeks after injection, with levels ranging from 2.31 × 10^3^ (heart) to 22.28 (spleen) copies/µg RNA. The data were explored graphically to evaluate potential differences between males and females without detecting major differences (Supplementary Figure S1).

Regarding the IV multiple-dose experiment, maximum values were observed at day 6 (after the second administration) and ranged from 1.12 × 10^6^ (heart) to 118.28 (liver) copies/µg RNA. Viral levels were only obtained at the last sampling time in the heart, lymph, and spleen, with values ranging from 8.59 × 10^4^ (spleen) to 5.27 × 10^3^ (lymph) copies/µg RNA after the administration of 10 doses (Supplementary Figure S2).

In the first subcutaneous administration experiment, viral levels ranged from 2.12 × 10^5^ (heart) to 54.10 (liver) copies/µg RNA at the first sampling time after two dose administrations. On day 24, after the 10^th^ dose, viral levels ranged from 3 × 10^3^ (lymph) to 4.3 (pancreas) copies/µg RNA (Supplementary Figure S3).

Given that sampling was sparse in the second SC multiple-dosing experiment, data were summarized as boxplots (Supplementary Figure S4). Maximum levels of viral copies/µg RNA were measured on day 24 in the spleen (1.44 × 10^3^ copies/µg RNA).

Supplementary Figure S5 represents both subcutaneous experiment concentration–time profiles.

### 3.2 Physiologically based pharmacokinetic model

For most of the organs, viral distribution was characterized using a perfusion-limited model assuming homogenous distribution within each of the organs; however, for the case of the muscle and heart, data were better described using a non-linear model for tissue uptake (*p* < 0.01). Models splitting organs in two or more compartments did not lead to a significant improvement in the fit (*p* > 0.05). The selected model resulted in a variant of the structure shown in Figure 2, as the parameters corresponding to the compartment lumping the rest of organs from which viral copies could not be estimated with sufficient precision and, therefore, were removed from the model.

**FIGURE 4:**
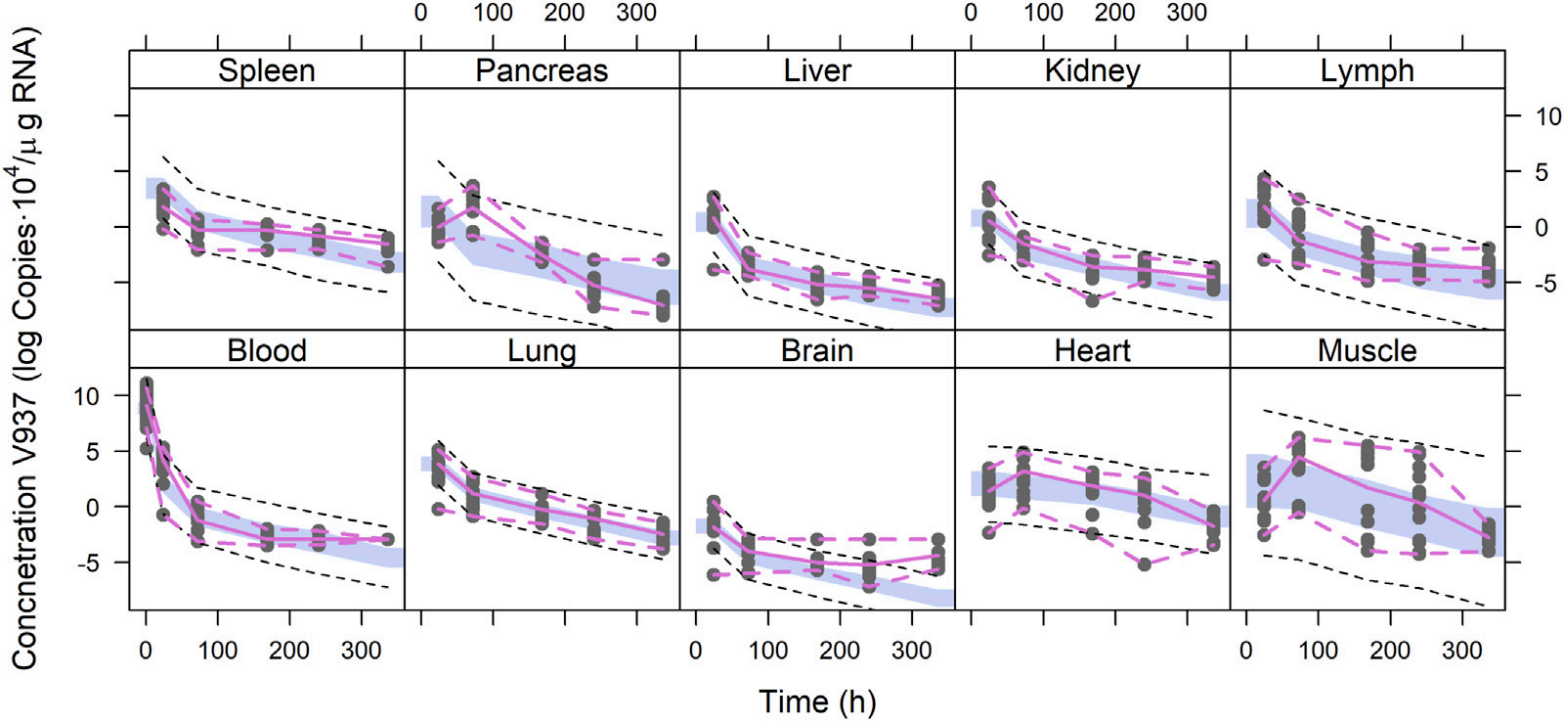
Simulation-based model diagnostics. Purple lines correspond to the 5th and 95th (dashed) and 50th (solid) percentiles of the raw data. The areas cover the 95% prediction intervals around the 50th percentiles calculated for each of the 1,000 simulated datasets. Black dashed lines correspond to the median of the 5th and 95th percentiles calculated for each of the 1,000 simulated datasets.

Figure 4 depicts the outcomes obtained from a simulation model diagnostic evaluation, which generally indicates that the model accurately captures both the typical trend and the variability in the observations over time. Despite the fact that more than one blood sample was taken from each animal, data did not support the estimation of inter-animal variability in any of the parameters of the model. Therefore, the degree of uncertainty used to generate the virtual studies and the concentration results shown in Figure 4 correspond to the magnitude of the residual variability, described using an additive error model on the logarithmic scale with estimates varying between 3.33 (muscle) and 1.01 (lung) log (copies/µg RNA). The estimates of the structural model parameters are listed in Table 1. In no case was the lower value of the 95% confidence interval calculated based on the log-likelihood profiles lower than or equal to zero, and therefore, the point estimates were considered precise.

**TABLE 1:** Parameter of estimates of the physiologically based pharmacokinetic model.

Organ/fluid	Partition coefficient	95% CI partition coefficient	Scaling factor (µg RNA/mL)	95% CI scaling factor	VMAX (copies/h)	95% CI VMAX
Blood	—	—	0.381	0.015–0.747	—	—
Brain	9.94 × 10^−3^	3.9 × 10^−4^–0.033	0.071	2.80 × 10^−3^–0.138	—	—
Heart	17.8	0.705–34.9	0.07	2.76 × 10^−3^–0.137	0.632	0.25–1.24
Kidney	8.39	0.33–16.5	5.15	0.20–13.2	—	—
Liver	60.9	2.41–119	188	7.45–483	—	—
Lung	548	21.7–1.07 × 10^−3^	15.1	0.59–29.6	—	—
Lymph	58.8	0.026–1.91	58.8	2.33–151	—	—
Muscle	218	8.63–427	3.93	0.16–13.2	31	1.23–60.8
Pancreas	45.7	1.81–89.6	20.6	0.82–52.9	—	—
Spleen	12.8	0.51–25.1	0.53	0.021–1.04	—	—

Total elimination clearance was estimated in 18.2 mL/h with a 95% confidence interval of 0.72–35.68 mL/h. Variants of the selected models considering the possibility that each organ could contribute to virus clearance did not improve the fit (*p* > 0.05). Results show a great degree of disparity in the estimates of the partition coefficients, ranging from the lowest value of 9.94 10^−3^ in the brain to the highest value of 548 in the lung. With respect to the scaling factors, estimates varied across the different tissues, although at a lesser degree compared to the partition coefficients. The derived values of the Michaelis–Menten constant for the muscle and heart were 0.568 and 0.038 copies/mL, respectively.

**FIGURE 5:**
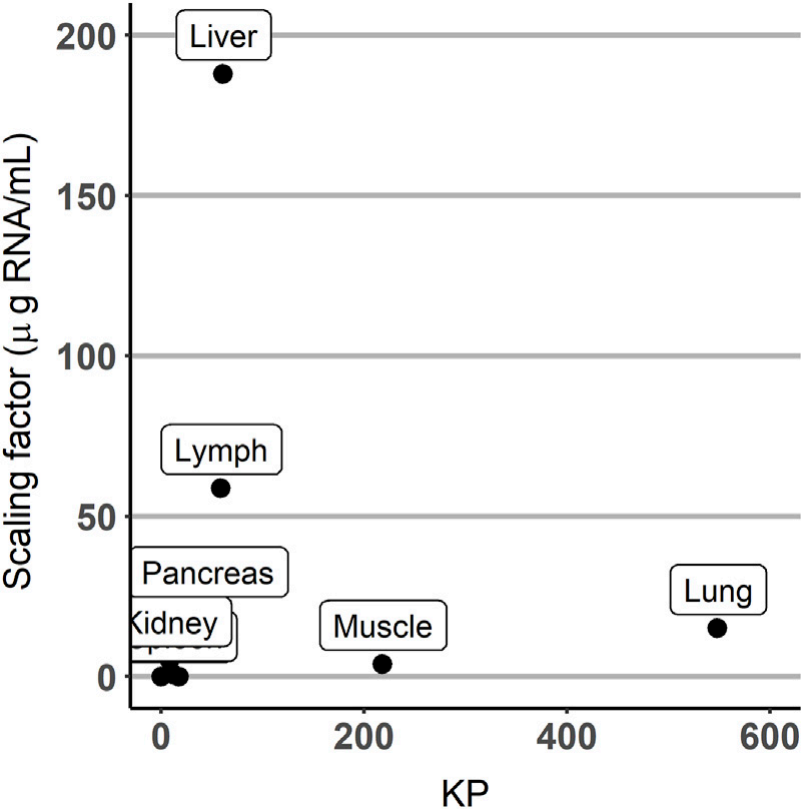
Scatterplot of the scaling factor vs. partition coefficient (KP) parameters.

The estimates of parameters of the partition coefficients and those corresponding to the scaling factors were not correlated, as shown in Figure 5.

A prolonged infusion was simulated to achieve a steady-state condition, allowing the comprehensive examination of the effects of these processes on the predicted organ level. In Supplementary Figure S6, the simulated 0–24 h concentration vs. time profiles in different organs is shown, assuming a 5 h continuous IV injection of 2.04 × 10^9^ TCID_50_. After a 5 h infusion, the decrease of virus concentration in the different organs of the body was observed. The uptake process is fast and similar for all organs except for the heart and muscle. The levels of copies in the heart and muscle are maintained after the end of the infusion time, the elimination of the virus occurring later than in the rest of the organs (Supplementary Figure S6A). Viral concentration in the heart begins to decline 10 h post-infusion initiation, while in the muscle tissue, the elimination of the virus commences 40 h after infusion initiation (Supplementary Figure S6B).

The results of the sensitivity analysis evaluation are shown in Supplementary Figure S8. Modulating the clearance parameter results in alterations of the AUC_0-tend_ across all organs, with the most significant impact observed in the lung AUC_0-tend_. Among the KP values, the most notable impacts on AUC_0-tend_ are observed when altering the KP values related to the lung and lymphatic system, influencing the AUC_0-tend_ of the lung and spleen, respectively.

### 3.3 Model validation

**FIGURE 6:**
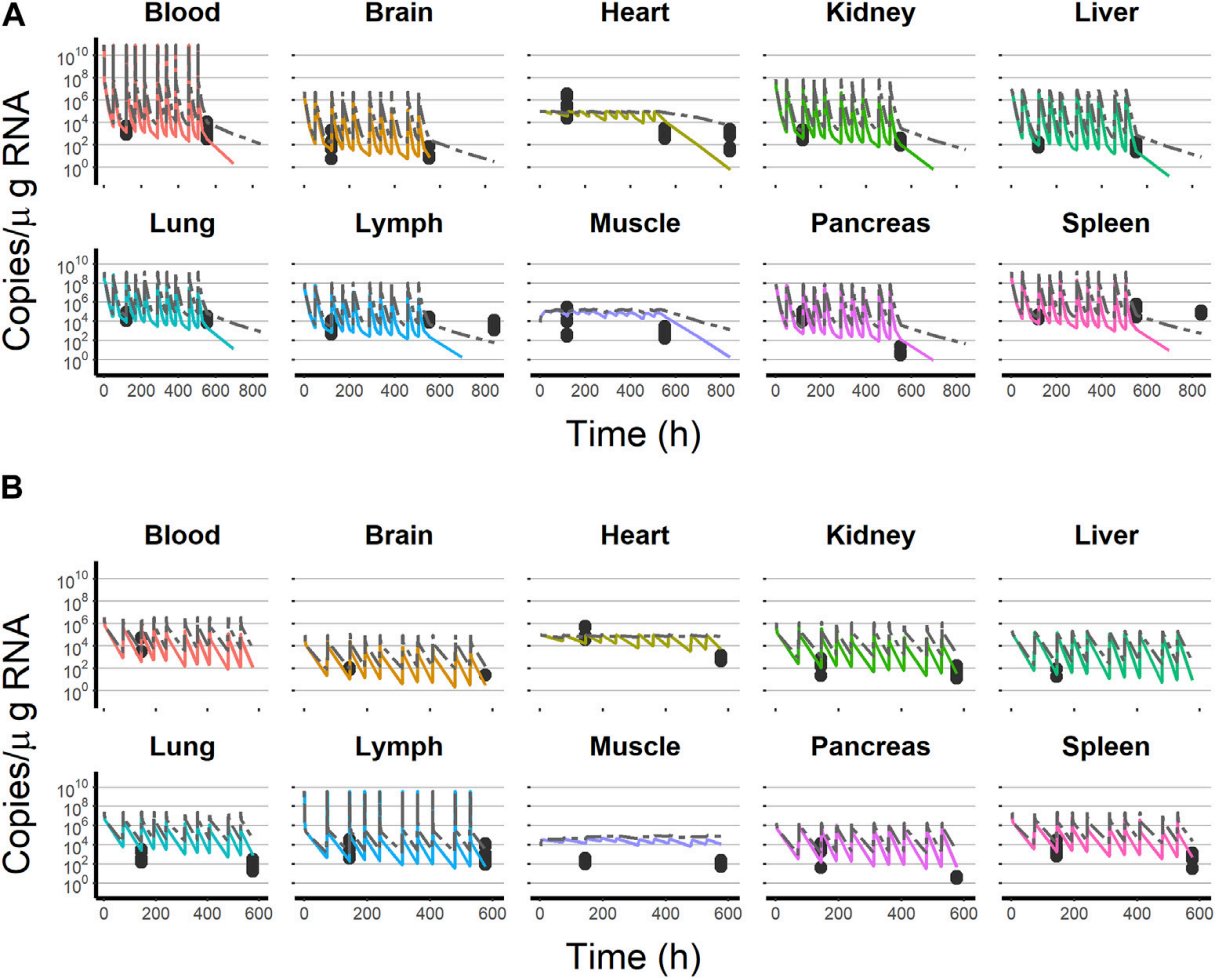
Final model simulation with (colored solid line) and without (dashed gray line) the time-variant clearance model vs. test dataset observations. **(A)** Intravenous administration experiment. **(B)** Subcutaneous administration experiment. Black dots represent test dataset observations. Solid colored lines represent the final model, including the time-variant clearance model. Each line color corresponds to an organ as follows: 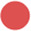
= blood, 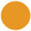
= brain, 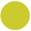
= heart, 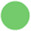
= kidney, 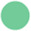
= liver, 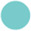
= lung, 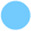
= lymph, 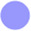
= muscle, 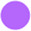
= pancreas, and 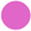
= spleen.

Results obtained during the initial steps of the validation procedure warranted additional model refinements/expansions. For example, when the selected model was used to simulate the blood and organ viral profiles during the IV multiple-dosing study, it was found that although the observed viral levels on day 6 after initiating the treatment were reasonably well captured, later measurements (day 36) were, in general, over-predicted by the model. The development of an immunogenicity response affecting viral elimination triggered by the continuous viral exposure consequence of the multiple-dosing schemas was considered and implemented empirically in the model as a time-varying clearance of the form CL = CL_0_ + θ_CL,T_ × time, where CL_0_ is the total clearance prior to the development of the immunogenic response and θ_CL,T_ represents the linear increase in CL with time (h). The time-variant clearance resulted in a significant reduction in the −2LL (−382). The estimate of θ_CL,T_ was 0.576 h^−1^ with a 95% confidence interval of 0.18–0.971 h^−1^, representing a 96.78 mL/h increase in CL per week of continuous viral exposure. Figure 6A shows that model-based predictions well match the observed viral copies for the multiple-dosing IV experiment. The figure also shows the predictions obtained from the time-invariant clearance.

After subcutaneous administration, a model considering the lymph node as the place receiving the viral dose and from which an instantaneous absorption (with an absolute value of bioavailability of 10%) occurs described the observed data of the multiple subcutaneous dosing experiment adequately (Figure 6B).

## 4 Discussion

The mechanism of action of oncolytic viruses has demonstrated some advantages over other oncological therapies, such as their selective capacity for tumor cells, the potential attainment of high exposures with relatively lower doses, or the capacity to neutralize the evasion of neoplastic cells (Russell et al., 2012; Goldufsky et al., 2013). Understanding the *in vivo* anticancer response of OVs represents an even greater challenge compared with other therapies used in oncology as the OV dynamics, namely, infectivity and viral replication, can affect its own pharmacokinetic exposure. In the current investigation, the disposition of V937 has been characterized in non-tumor-bearing Hu/Mu ICAM-1 transgenic mice developing a physiologically based pharmacokinetic model. As viral infection and replication occur in tumor, using non-tumor-bearing Hu/Mu ICAM-1 transgenic mice enable us to characterize the pharmacokinetics of OV without the interfering impact of its own replication. The model described longitudinal virus exposure in 10 different organs and fluids and was validated with sets of data gathered under different scenarios with regard to dose levels, dosing regimens, and route of administration.

PBPK models of large molecules have been published, where organs were divided into endosomal, vascular, and interstitial spaces (three sub-compartments), as in the case of monoclonal antibodies (Davda et al., 2008; Shah and Betts, 2012; Khot et al., 2017; Niederalt et al., 2018), or only into two compartments (vascular and interstitial) to characterize, for example, the biodistribution of T cells (Khot et al., 2019). These organ compartmentalizations were initially considered, but given the lack of physiological models for OV, a simpler structure with fewer assumptions regarding extravasation, vascular, or lymphatic reflection coefficients was developed as an important first step. The data-driven model-building approach used in the current analysis supported this simpler structure based on the perfusion-limited model, therefore assuming homogeneous viral distribution within each organ. In accordance with the limitation imposed by the scarcity of available data, it was assumed that concentrations in the peripheral veins are equal to the blood compartment since no observations were specifically measured in arteries.

**FIGURE 7:**
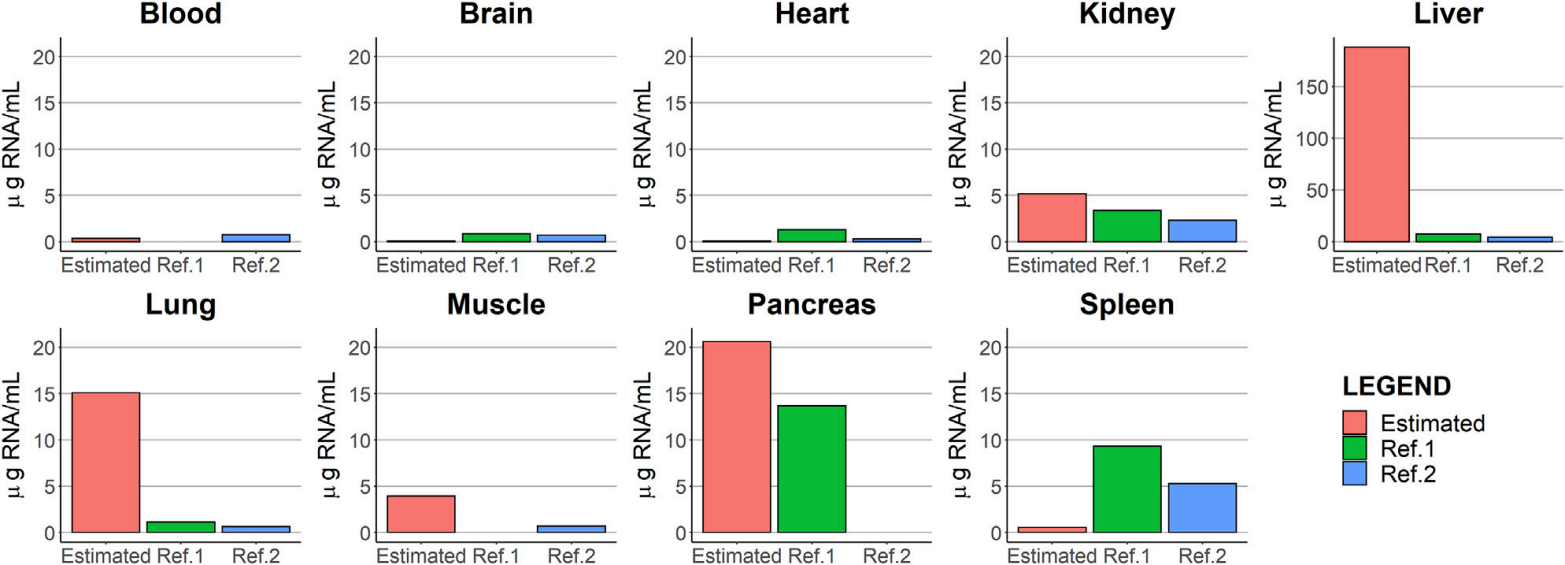
Comparison between the parameter estimates of the scaling factors [RNA (µg RNA/mL)] and those values obtained from two different literature sources, Brisco et al. (1997) and Krawiec et al. (2009).

One of the challenges encountered during the analysis was that the dose was expressed in units of TCID_50_ of V937, whereas observations were reported as copies/µg RNA of V937. To maintain the consistency of units, viral copies had to be scaled by the (non-available) organ-specific amount of RNA. First, values of the amount of RNA from the literature were used; however, some predictions were far from the observed values. Furthermore, the measures obtained from the literature are susceptible to error and exhibit substantial inter-experimental variability. Then, those scaling factors were estimated as parameters in the model together with the partition coefficients, providing, in this case, an adequate description of the viral exposure. The comparison of the estimated scaling factor RNA values (µg RNA/mL) with those reported in the literature is represented in Figure 7. The estimated parameters were similar to values from references in the blood, brain, heart, kidney, muscle, and pancreas. The spleen estimated scaling factor differed by an order of magnitude from literature data. For the liver and lung, the estimated values differed largely from those reported in the literature. No RNA data were found in the lymph node in mice. The absence of information on the amount of RNA present in the different organs beyond the references found does not allow us to elucidate what may be occurring in the organs with the highest level of RNA estimates.

A proper and complete data collection would have allowed overcoming this limitation without the need to carry out additional experiments. Therefore, good communication between modelers and experimentalists is important to obtain measurements that enable a better characterization of the molecule under study.

The partition coefficient in the lung was estimated to be the highest across the different organs studied (KP = 548), a result consistent with the ability of the virus to infect the respiratory tract (McCarthy et al., 2019). In contrast, the brain showed the lowest partition coefficient, partly explained by the fact that it represents a site less susceptible to virus infection. V937 RNA levels in the muscle and heart increases at least up to day 3 after a single IV dose administration, achieving levels approximately 15–20-fold and 5-fold, higher with respect to the values observed at day 1, respectively. Those concentration vs. time profiles differed from the rest of the organs and were successfully captured using a non-linear mechanism. The possibility that the Michaelis–Menten input mechanism was the result of the lower expression of the ICAM-1, the receptor responsible for the entry of the virus into cells (Johansson et al., 2004; Au et al., 2007; Berry et al., 2008; Annels et al., 2018; 2019), has been initially discharged as in-house data revealed that ICAM-1 expression in those two tissues was not different from the rest (data not shown). Furthermore, during the model-building process, different approaches to consider the potential impact of ICAM-1 expression on viral distribution were explored. However, there was no correlation between the concentration of ICAM-1 and partition coefficients or organ viral levels (Supplementary Figure S7), suggesting that although the receptor is needed for internalization, it might not be the limiting factor.

An intense sampling during short times after administration is required to better characterize tissue distribution (Annels et al., 2019), a disposition process that, in the particular case of OVs, is difficult to decouple from replication. Although the current study was performed in non-tumor-bearing organs and replication was not identified, the complete absence of replication cannot be ruled out. A recent preclinical experiment using methods to discriminate between viral distribution and viral replication showed that the latter process occurred in healthy tissues but was restricted to those with resident macrophages (Dambra et al., 2023). This could suggest that macrophages play a role in shaping viral dynamics. This assumption is substantiated by several studies indicating that macrophages can exert an influence on the suppression of the virus through type I interferon (Aichele et al., 2003; Honke et al., 2012), thereby limiting the dissemination of the virus to the organs (Cervantes-barragán et al., 2013) influencing its biodistribution. In fact, previous findings pinpoint the possibility that the target receptor does not play a major role in tissue distribution (Campbell and Kahl, 1989; Fazakerley et al., 1991). Furthermore, the function of resident liver macrophages (Kupffer cells) in detecting viruses and eliciting an antiviral response has been previously documented (Lang et al., 2010; Lang and Lang, 2015). This leads us to postulate that the elevated liver scaling factor (188 µg RNA/mL) may be associated with the large number of macrophages within the liver reported in prior studies (SZU-HEE LEE, PHYLLIS M. SZU-HEE et al., 1985; Liu et al., 2019). These types of experiments can be of great interest to adequately characterize viral kinetics and the impact of viral replication on exposure. Similar to the liver, the spleen is recognized for its role in the filtration of pathogenic agents and exogenous substances. However, the data reveal that a maximum of 1.44 × 10^3^ copies/µg RNA is reached during the final sampling period. This accumulation of V937 in the spleen may be attributed to the delayed activation of the antiviral response in this particular tissue, as suggested by the previous literature (Dambra et al., 2023).

Virus clearance from the circulatory system was assumed as it has been included in previous models for oncolytic viruses. This elimination of V937 in the systemic circulation occurs through macrophages and dendritic cells and not mainly by metabolism (Russell and Peng, 2007; Rojas et al., 2016; Tan et al., 2017). The rapid drop in viral load on day 1 is reflected by a high estimate of total clearance (18.2 mL/h). This value aligns with the clearance rate previously estimated in a prior analysis of just the blood samples, which was identified as 21.2 mL/h. In addition, this result is consistent with other studies reporting short values of half-life (Garcia-Carbonero et al., 2017; Béguin et al., 2021; Parra-Guillen et al., 2021). Development of an immunogenic reaction against biological therapies is a known fact that it is reflected by an increase in CL with time (Béguin et al., 2021; Parra-Guillen et al., 2021; Dambra et al., 2023), as has been the case in the current investigation. As a representative instance, the study by Dambra *et al.* divided the viral kinetics into early and late time points, with the clearance rate estimated to be 21.8 and 282.8 mL/h, respectively. In line with this, our study determined an initial clearance value of 18.2 mL/h and observed a weekly increase of 98 mL/h in clearance. However, the estimate of the parameter accounting for the increase in CL over time has to be interpreted with caution since viral copies were measured on just two occasions during the multiple-dosing regimens. Whether this immunogenic reaction has the potential to be translated into an immune tumor response needs to be specifically addressed in future investigations.

During the validation exercise using data from the subcutaneous administration, the model was modified to incorporate an absorption process taken place by the lymph, as it collects large molecules, such as monoclonal antibodies or viruses (Davis and Bugelski, 1998). Given the lack of data after early time points, instantaneous absorption into lymph was assumed, thus representing a limitation. The estimate of the absolute bioavailability was low (10%), a value in accordance with that reported for other large molecules (Al Shoyaib et al., 2020; Jaradat et al., 2020).

## 5 Conclusion

In summary, a PBPK model has been developed for the oncolytic virus V937, being, to our knowledge, the first PBPK model developed to characterize OV biodistribution. The model was able to describe *in vivo* data successfully and was subsequently validated using a test dataset, including other routes of administration. Thus, the model provides a quantitative understanding of the elimination and tissue uptake processes of V937 and can help to leverage and interpret systemic exposure PCR data obtained both from non-clinical and clinical studies. The utilization of this model as a reference point and potential for future expansion is crucial. The subsequent step would entail characterizing exposure in an animal model exhibiting a tumor in order to evaluate the influence of viral dynamics. Additionally, the model is susceptible to being scaled-up to humans by adjusting the physiological-related parameters and using allometric scaling with respect to elimination clearance. In addition, these data and modeling exercises help to leverage systemic exposure in humans, especially with respect to distribution.

## Data Availability

The original contributions presented in the study are included in the article/Supplementary Material; further inquiries can be directed to the corresponding author.
