# Supplementary material for: A physiologically based pharmacokinetic model for V937 oncolytic virus in mice
Source: Front Pharmacol. 2023 Sep 13;14:1211452. doi: 10.3389/fphar.2023.1211452 (PMC10524596; doi:10.3389/fphar.2023.1211452)
Supplement: Supplementary file 1 [file DataSheet3.PDF]

```
;; 1. Based on: 845

;; x1. Author: sara96


$PROB WB-PBPK-V937

$INPUT ID TIME AAMT AMT DVV LOGDVV DV2 DV CMT MDV EVID SEX WT CRCL LQQ LOGLQQ
LQ2 LQ BLQ

$DATA dataset015nmloglowLY.csv IGNORE=@

$SUBROUTINES ADVAN13 TOL=9

$MODEL

;;----------------------------PBPK Compartments---------------------------;;

COMP = (ART) ; 1- Arteries

COMP = (VEN) ; 2- Veins

COMP = (LUN) ; 3- Lungs

COMP = (BRA) ; 4- Brain

COMP = (HRT) ; 5- Heart vasc

COMP = (MUS) ; 6- Muscle

COMP = (SPL) ; 7- Spleen

COMP = (PAN) ; 8- Pancreas

COMP = (LIV) ; 9- Liver

COMP = (KID) ; 10-Kidneys

COMP = (RES) ; 11-Rest

COMP = (LYM) ; 12-Lymph node



;;----------------------------PBPK--------------------------------------;;

;; ------------------------ Tissue volumes (mL) ------------------------- ;;

$PK

VART = 0.228 ; Arterial volume (mL)

VVEN = 0.524 ; Venous volume (mL)

VLUN = 0.1 ; Lungs weight (Kg) ICRP

VBRA = 0.170 ; Brain volume (mL)
```

VHRT = 0.095 ; Heart volume (mL) ICRP

VSPL = 0.1 ; Spleen volume (mL) ICRP

VPAN = 0.13; Pancreas volume (mL)ICRP

VHEP = 1.30 ; Hepatic volume (mL) ICRP

VKID = 0.340 ; Kidney volume (mL) ICRP

VRES = 6.9; Rest of body volume bones ICRP

VLYM = 0.113; lymph volume

VMUS = 10 ; Total Muscle volume (mL) ICRP

;; --------------------------- Blood flows (mL/H) ------------------------- ;;

CO = 328.2; Cardiac output in mL/h

QART = CO ; Artery Blood flow

QVEN = CO ; Venous blood flow

QLUN = CO ; Lung blood flow

QBRA = 7.8; Brain blood flow

QHRT = 16.8 ; Heart blood flow

QMUS = 54.6 ; Muscle blood flow

QSPL = 5.4 ; Spleen blood flow

QPAN = 3.12 ; Pancreas blood flow

QKID = 78 ; Kidney blood flow

QHEPA= 21; Hepatic arterial blood flow

QRES = 139.83; Rest of Body blood flow

QLYM = 1.65 ; Lymph flow rate

QHEPT= QHEPA + QSPL + QPAN; Total hepatic blood flow HEPA+SPL+PAN)

;-----------------------------Lymph flows (ml/H) --------------------------------

LLUN = QLUN/500

LBRA = QBRA/500

```
LHRT = QHRT/500

LMUS = QMUS/500

LSPL = QSPL/500

LPAN = QPAN/500

LKID = QKID/500

LHEPT= QHEPA/500

LRES = QRES/500


;; --------------------------- Clearance parameters ---------------------------

TVCL= THETA(1) ; CL

CL= TVCL*EXP(ETA(1))


;; -------------------------- Partition Coefficients --------------------------


KLUN = THETA(2)*EXP(ETA(2))

KBRA = THETA(3)*EXP(ETA(2))

KHRT = THETA(4)*EXP(ETA(2))

KMUS = THETA(5)*EXP(ETA(2))

KSPL = THETA(6)*EXP(ETA(2))

KPAN = THETA(7)*EXP(ETA(2))

KHEP = THETA(8)*EXP(ETA(2))

KKID = THETA(9)*EXP(ETA(2))

KRES = THETA(10)*EXP(ETA(2))

KLYM = THETA(11)*EXP(ETA(2))


;-------------------------- SCALING FACTORS --------------------------

RNA_BLOOD=THETA(12)*EXP(ETA(3))

RNA_LUN=THETA(13)*EXP(ETA(3))

RNA_BRA=THETA(14)*EXP(ETA(3))

RNA_HRT=THETA(15)*EXP(ETA(3))

RNA_MUS=THETA(16)*EXP(ETA(3))
```

```
RNA_SPL=THETA(17)*EXP(ETA(3))
RNA_PAN=THETA(18)*EXP(ETA(3))
RNA_HEP=THETA(19)*EXP(ETA(3))
RNA_KID=THETA(20)*EXP(ETA(3))
RNA_LYM=THETA(21)*EXP(ETA(3))

S1=VART*RNA_BLOOD
S2=VVEN*RNA_BLOOD
S3=VLUN*RNA_LUN
S4=VBRA*RNA_BRA
S5=VHRT*RNA_HRT
S6=VMUS*RNA_MUS
S7=VSPL*RNA_SPL
S8=VPAN*RNA_PAN
S9=VHEP*RNA_HEP
S10=VKID*RNA_KID
S12=VLYM*RNA_LYM

VMAXM = THETA(22)*EXP(ETA(4))
VMAXH = THETA(23)*EXP(ETA(4))

$DES
;; ------------------------- Compartment concentrations -----------------------
;;
C1 = A(1)/VART ; AMT/Arterial volume
C2 = A(2)/VVEN ; AMT/Venous volume
C3 = A(3)/VLUN ; AMT/Lung volume
C4 = A(4)/VBRA ; AMT/Brain volume
C5 = A(5)/VHRT ; AMT/Heart volume
C6 = A(6)/VMUS ; vascular cmt muscle
C7 = A(7)/VSPL ; AMT/Spleen volume
```

```
C8 = A(8)/VPAN ; AMT/Pan volume V

C9 = A(9)/VHEP ; AMT/Hepatic volume

C10= A(10)/VKID ; AMT/Kidney volume

C11= A(11)/VRES ; AMT/Rest volume

C12= A(12)/VLYM ;


;; ------------------------------- Abbreviations ------------------------------


KMM = VMAXM/QMUS

KMH = VMAXH/QHRT

VENIN1 = ((QBRA-LBRA)*C4/KBRA)+((QHRT-LHRT)*C5/KHRT)+((QMUS-
LMUS)*C6/KMUS)+(QLYM*C12/KLYM)

VENIN2 = ((QHEPT-LHEPT)*C9/KHEP)+((QKID-LKID)*C10/KKID)+((QRES-LRES)*C11/KRES)

VENOUT = (QLUN*C2)+(C2*CL)

HEPIN = (C1*QHEPA)+((QSPL-LSPL)*C7/KSPL)+ ((QPAN-LPAN)*C8/KPAN)

HEPOUT = ((QHEPT-LHEPT)*C9/KHEP)+(C9*LHEPT)



;;-----------------------------------PBPK ODES---------------------------------------

DADT(1) = ((QLUN-LLUN)*C3/KLUN)-(QART*C1)

DADT(2) = VENIN1+VENIN2-VENOUT

DADT(3) = (QVEN*C2)-((QLUN-LLUN)*C3/KLUN)-(C3*LLUN)

DADT(4) = (QBRA*C1)-((QBRA-LBRA)*C4/KBRA)-(C4*LBRA)

DADT(5) = ((VMAXH*C1)/(KMH + C1))-((QHRT-LHRT)*C5/KHRT)-(C5*LHRT)

DADT(6) = ((VMAXM*C1)/(KMM + C1))-((QMUS-LMUS)*C6/KMUS)-(C6*LMUS)

DADT(7) = (QSPL*C1)-((QSPL-LSPL)*C7/KSPL)-(C7*LSPL)

DADT(8) =(QPAN*C1)-((QPAN-LPAN)*C8/KPAN)-(C8*LPAN)

DADT(9)= HEPIN-HEPOUT

DADT(10)= (QKID*C1)-((QKID-LKID)*C10/KKID)-(C10*LKID)

DADT(11)= (QRES*C1)-((QRES-LRES)*C11/KRES)-(C11*LRES)
```

DADT(12)=
(C3*LLUN)+(C4*LBRA)+(C5*LHRT)+(C6*LMUS)+(C7*LSPL)+(C8*LPAN)+(C9*LHEPT)+(C10*LKID)+(C11*LRES)-(QLYM*C12/KLYM)

$ERROR

AA1 = A(1) ; AMT/Arteries

AA2 = A(2) ; AMT/Veins

AA3 = A(3) ; AMT/Lungs

AA4 = A(4) ; AMT/Brain

AA5 = A(5) ; AMT/Heart

AA6 = A(6) ; AMT/Muscle VAS

AA7 = A(7) ; AMT/Spleen EX

AA8 = A(8) ; AMT/Spleen

AA9 = A(9) ; AMT/Pancreas VAS

AA10= A(10) ; AMT/Pancreas EX

AA11= A(11) ; AMT/ Liver

AA12= A(12) ; AMT/Kidney

CC1 = A(1)/S1; AMT/Arterial volume

CC2 = A(2)/S2; AMT/Venous volume

CC3 = A(3)/S3; AMT/Lung volume

CC4 = A(4)/S4 ; AMT/Brain volume

CC5 = A(5)/S5 ; AMT/Heart volume

CC6 = A(6)/S6 ; AMT/Muscle volume

CC7 = A(7)/S7; AMT/Spleen volume

CC8 = A(8)/S8 ; AMT/Pancreas volume

CC9 = A(9)/S9 ; AMT/Hepatic volume

CC10= A(10)/S10 ; AMT/Kidney volume

CC11= A(11)/VRES ; AMT/Rest volume

CC12= A(12)/S12 ; AMT/LYMPH

```
IF (CMT.EQ.2.AND.BLQ.EQ.0) THEN
F_FLAG=0
IPRD2 = CC2
IPRED = LOG(IPRD2+1E-6)
IRES = DV-IPRED   ;residuals
WVEN = THETA(24)  ; additive error
IWRES = IRES/WVEN  ;residuals/error
Y = IPRED + WVEN*EPS(1)      ;
ENDIF
IF (CMT.EQ.2.AND.BLQ.EQ.1) THEN
F_FLAG=1
IPRD2 = CC2
IPRED = LOG(IPRD2+1E-6)
IRES = DV-IPRED   ;residuals
WVEN = THETA(24)  ; additive error
IWRES = IRES/WVEN  ;residuals/error
Y=PHI((LQ-IPRED)/WVEN) + 1E-6
ENDIF


IF (CMT.EQ.3.AND.BLQ.EQ.0) THEN
F_FLAG=0
IPRD2 = CC3
IPRED = LOG(IPRD2+1E-6)
IRES = DV-IPRED   ;residuals
WLUN = THETA(25)  ; additive error
IWRES = IRES/WLUN  ;residuals/error
Y = IPRED + WLUN*EPS(1)      ;
ENDIF
IF (CMT.EQ.3.AND.BLQ.EQ.1) THEN
```

```
F_FLAG=1
IPRD2 = CC3
IPRED = LOG(IPRD2+1E-6)
IRES = DV-IPRED   ;residuals
WLUN = THETA(25)  ; additive error
IWRES = IRES/WLUN  ;residuals/error
Y=PHI((LQ-IPRED)/WLUN) + 1E-6
ENDIF


IF (CMT.EQ.4.AND.BLQ.EQ.0) THEN
F_FLAG=0
IPRD2 = CC4
IPRED = LOG(IPRD2+1E-6)
IRES = DV-IPRED   ;residuals
WBRA = THETA(26)  ; additive error
IWRES = IRES/WBRA  ;residuals/error
Y = IPRED + WBRA*EPS(1)      ;
ENDIF
IF (CMT.EQ.4.AND.BLQ.EQ.1) THEN
F_FLAG=1
IPRD2 = CC4
IPRED = LOG(IPRD2+1E-6)
IRES = DV-IPRED   ;residuals
WBRA = THETA(26)  ; additive error
IWRES = IRES/WBRA  ;residuals/error
Y=PHI((LQ-IPRED)/WBRA) + 1E-6
ENDIF


IF (CMT.EQ.5.AND.BLQ.EQ.0) THEN
```

```
F_FLAG=0
IPRD2 = CC5
IPRED = LOG(IPRD2+1E-6)
IRES = DV-IPRED   ;residuals
WHRT = THETA(27)  ; additive error
IWRES = IRES/WHRT  ;residuals/error
Y = IPRED + WHRT*EPS(1)
ENDIF
IF (CMT.EQ.5.AND.BLQ.EQ.1) THEN
F_FLAG=1
IPRD2 = CC5
IPRED = LOG(IPRD2+1E-6)
IRES = DV-IPRED   ;residuals
WHRT = THETA(27)  ; additive error
IWRES = IRES/WHRT  ;residuals/error
Y=PHI((LQ-IPRED)/WHRT) + 1E-6
ENDIF


IF (CMT.EQ.6.AND.BLQ.EQ.0) THEN
F_FLAG=0
IPRD2 = CC6
IPRED = LOG(IPRD2+1E-6)
IRES = DV-IPRED   ;residuals
WMUS = THETA(28)  ; additive error
IWRES = IRES/WMUS  ;residuals/error
Y = IPRED + WMUS*EPS(1)
ENDIF
IF (CMT.EQ.6.AND.BLQ.EQ.1) THEN
F_FLAG=1
IPRD2 = CC6
```

```
IPRED = LOG(IPRD2+1E-6)

IRES = DV-IPRED   ;residuals

WMUS = THETA(28)  ; additive error

IWRES = IRES/WMUS  ;residuals/error

Y=PHI((LQ-IPRED)/WMUS) + 1E-6

ENDIF



IF (CMT.EQ.7.AND.BLQ.EQ.0) THEN

F_FLAG=0

IPRD2 = CC7

IPRED = LOG(IPRD2+1E-6)

IRES = DV-IPRED   ;residuals

WSPL = THETA(29)  ; additive error

IWRES = IRES/WSPL  ;residuals/error

Y = IPRED + WSPL*EPS(1)

ENDIF

IF (CMT.EQ.7.AND.BLQ.EQ.1)  THEN

F_FLAG=1

IPRD2 = CC7

IPRED = LOG(IPRD2+1E-6)

IRES = DV-IPRED   ;residuals

WSPL = THETA(29)  ; additive error

IWRES = IRES/WSPL  ;residuals/error

Y=PHI((LQ-IPRED)/WSPL) + 1E-6

ENDIF



IF (CMT.EQ.8.AND.BLQ.EQ.0) THEN

F_FLAG=0

IPRD2 = CC8
```

```
IPRED = LOG(IPRD2+1E-6)
IRES = DV-IPRED   ;residuales
WPAN = THETA(30)  ; additive error
IWRES = IRES/WPAN  ;residuales/error
Y = IPRED + WPAN*EPS(1)
ENDIF
IF (CMT.EQ.8.AND.BLQ.EQ.1) THEN
F_FLAG=1
IPRD2 = CC8
IPRED = LOG(IPRD2+1E-6)
IRES = DV-IPRED   ;residuals
WPAN = THETA(30)  ; additive error
IWRES = IRES/WPAN ;residuals/error
Y=PHI((LQ-IPRED)/WPAN) + 1E-6
ENDIF


IF (CMT.EQ.9.AND.BLQ.EQ.0) THEN
F_FLAG=0
IPRD2 = CC9
IPRED = LOG(IPRD2+1E-6)
IRES = DV-IPRED   ;residuals
WHEP = THETA(31)  ;additive error
IWRES = IRES/WHEP  ;residuals/error
Y = IPRED + WHEP*EPS(1)
ENDIF
IF (CMT.EQ.9.AND.BLQ.EQ.1) THEN
F_FLAG=1
IPRD2 = CC9
IPRED = LOG(IPRD2+1E-6)
IRES = DV-IPRED   ;residuals
```

```
WHEP = THETA(31)  ;additive error
IWRES = IRES/WHEP  ;residuals/error
Y=PHI((LQ-IPRED)/WHEP) + 1E-6
ENDIF


IF (CMT.EQ.10.AND.BLQ.EQ.0) THEN
F_FLAG=0
IPRD2 = CC10
IPRED = LOG(IPRD2+1E-6)
IRES = DV-IPRED   ;residuals
WKID = THETA(32)  ;additive error
IWRES = IRES/WKID  ;residuals/error
Y = IPRED + WKID*EPS(1)
ENDIF
IF (CMT.EQ.10.AND.BLQ.EQ.1) THEN
F_FLAG=1
IPRD2 = CC10
IPRED = LOG(IPRD2+1E-6)
IRES = DV-IPRED   ;residuals
WKID = THETA(32)  ; additive error
IWRES = IRES/WKID ;residuals/error
Y=PHI((LQ-IPRED)/WKID) + 1E-6
ENDIF

IF (CMT.EQ.12.AND.BLQ.EQ.0) THEN
F_FLAG=0
IPRD2 = CC12
IPRED = LOG(IPRD2+1E-6)
IRES = DV-IPRED   ;residuals
WKID = THETA(33)  ; additive error
```

```
IWRES = IRES/WKID  ;residuals/error
Y = IPRED + WKID*EPS(1)
ENDIF
IF (CMT.EQ.12.AND.BLQ.EQ.1) THEN
F_FLAG=1
IPRD2 = CC12
IPRED = LOG(IPRD2+1E-6)
IRES = DV-IPRED   ;residuals
WKID = THETA(33)  ; additive error
IWRES = IRES/WKID ;residuals/error
Y=PHI((LQ-IPRED)/WKID) + 1E-6
ENDIF



;; ---------------------------- Initial estimates Theta PBPK ----------------------------- ;;

$THETA
(0, 7.04) ;1 ~CL
(0, 436) ;2 ~KLUN
(0, 0.36) ;3 ~KBRA
(0, 100) ;4 ~KHRT
(0, 168) ;5 ~KMUS
(0, 345) ;6 ~KSPL
(0, 58) ;7 ~KPAN
(0, 5.56) ;8~KHEP
(0, 12.8) ;9~KKID
(0, 35) ;10~KRES
(0, 1) ;10~KLYM
(0, 0.746) ;12~RNA_BLOOD
(0, 37.6) ;13~RNA_LUN
```

```
(0, 7) ;14~RNA_BRA

(0, 6.98) ;15~RNA_HRT

(0, 7) ;16~RNA_MUS

(0, 53) ;17~RNA_SPL

(0, 2060) ;18~RNA_PAN

(0, 44) ;19~RNA_HEP

(0, 23) ;20~RNA_KID

(0, 1) ;21~RNA_LYM

(0, 20.3) ;22~VMAXM

(0, 2.18) ;23~VMAXH

(0, 1.43) ;24~WVEN

(0, 0.995) ;25~WLUN

(0, 1.06) ;26~WBRA

(0, 1.76) ;27~WHRT

(0, 3.32) ;28~WMUS

(0, 1.63) ;29~WSPL

(0, 2.28) ;30~WPAN

(0, 1.68) ;31~WHEP

(0, 1.22) ;32~WKID

(0, 1.22) ;33~WLYM


$OMEGA 0 FIX  ;1 ~IIV_CL

$OMEGA 0 FIX  ;2 ~IIV_KP

$OMEGA 0 FIX  ;3 ~IIV_RNA

$OMEGA 0 FIX  ;4 ~IIV_RNA


; ----------------------------- SIGMA --------------------------- ;;


$SIGMA 1 FIX  ;1~ Prop RES_ERR
```

$ESTIMATION METHOD=1 INTER LAPLACIAN POSTHOC NOHABORT PRINT=5 MAXEVALS=9999

$COV PRINT=E

$TABLE ID TIME CC1 CC2 CC3 CC4 CC5 CC6 CC7 CC8 CC9 CC10 CC11 CC12 IPRED IWRES EVID DV CMT CWRES NOPRINT ONEHEADER FILE=sdtab845

$TABLE ID KLUN KBRA KHRT KMUS KSPL KPAN KHEP KKID KRES KLYM VMAXM VMAXH RNA_BLOOD RNA_LUN RNA_BRA RNA_HRT RNA_MUS RNA_SPL RNA_PAN RNA_HEP RNA_KID RNA_LYM CL NOPRINT ONEHEADER FILE=patab845
