# Supplementary material for: A physiologically based pharmacokinetic model for V937 oncolytic virus in mice
Source: Front Pharmacol. 2023 Sep 13;14:1211452. doi: 10.3389/fphar.2023.1211452 (PMC10524596; doi:10.3389/fphar.2023.1211452)
Supplement: Supplementary file 2 [file DataSheet2.docx]

Supplementary Material

A physiologically-based pharmacokinetic model for V937 oncolytic virus in mice

**Sara Peribañez-Dominguez, Zinnia P Parra-Guillen, Tomoko Freshwater, Iñaki F Troconiz**

*** Correspondence:** Zinnia P Parra-Guillen: [zparra@unav.es](mailto:zparra@unav.es)

# Supplementary Figures and Tables

**Supplementary Table I**. Values of the Physiological parameters used to develop the physiologically-based pharmacokinetic model.

| **Organ** | **Organ volume** | **Blood flow** | **Lymph flow** |
| --- | --- | --- | --- |
|  | **(mL/kg)** | **(mL/h)** | **(mL/h)** |
| **Arteries** | 11.4 | 328.2 |  |
| **Brain** | 8.5 | 7.8 | 0.016 |
| **Heart** | 4.75 | 16.8 | 0.034 |
| **Kidney** | 17 | 78 | 0.156 |
| **Liver** | 65 | 21 | 0.042 |
| **Lung** | 5 | 328.2 | 0.652 |
| **Lymph** | 5.65 | 1.65 |  |
| **Muscle** | 500 | 54.6 | 0.109 |
| **Pancreas** | 6.5 | 3.12 | 0.006 |
| **Rest** | 345 | 139.83 | 0.280 |
| **Spleen** | 5 | 5.4 | 0.011 |
| **Veins** | 26.2 | 328.2 |  |


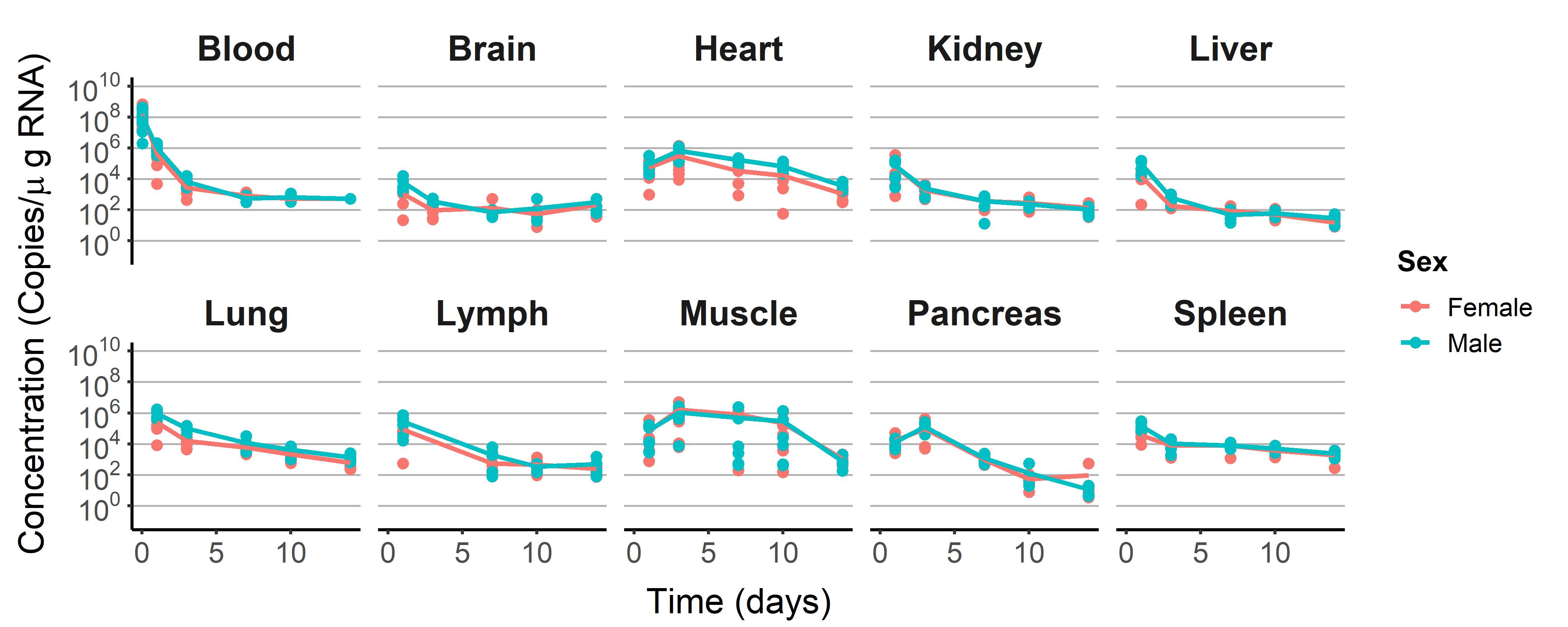


**Supplementary Figure 1. Training dataset concentration-time profiles.** Dots represent the observations. Solid lines represents median tendency in each organ coloured by sex.


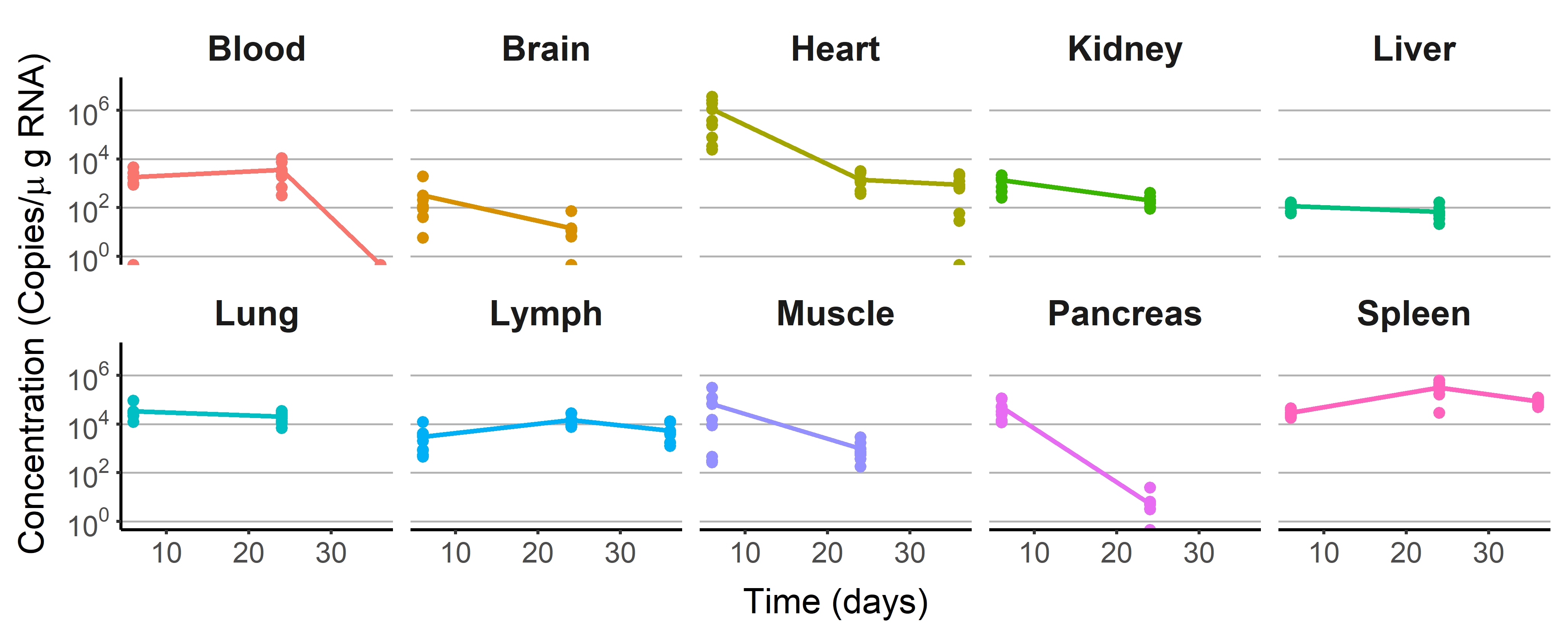


**Supplementary Figure 2.** **IV experiment concentration-time profiles.** Dots represent the observations. Solid lines represents median tendency in each organ.


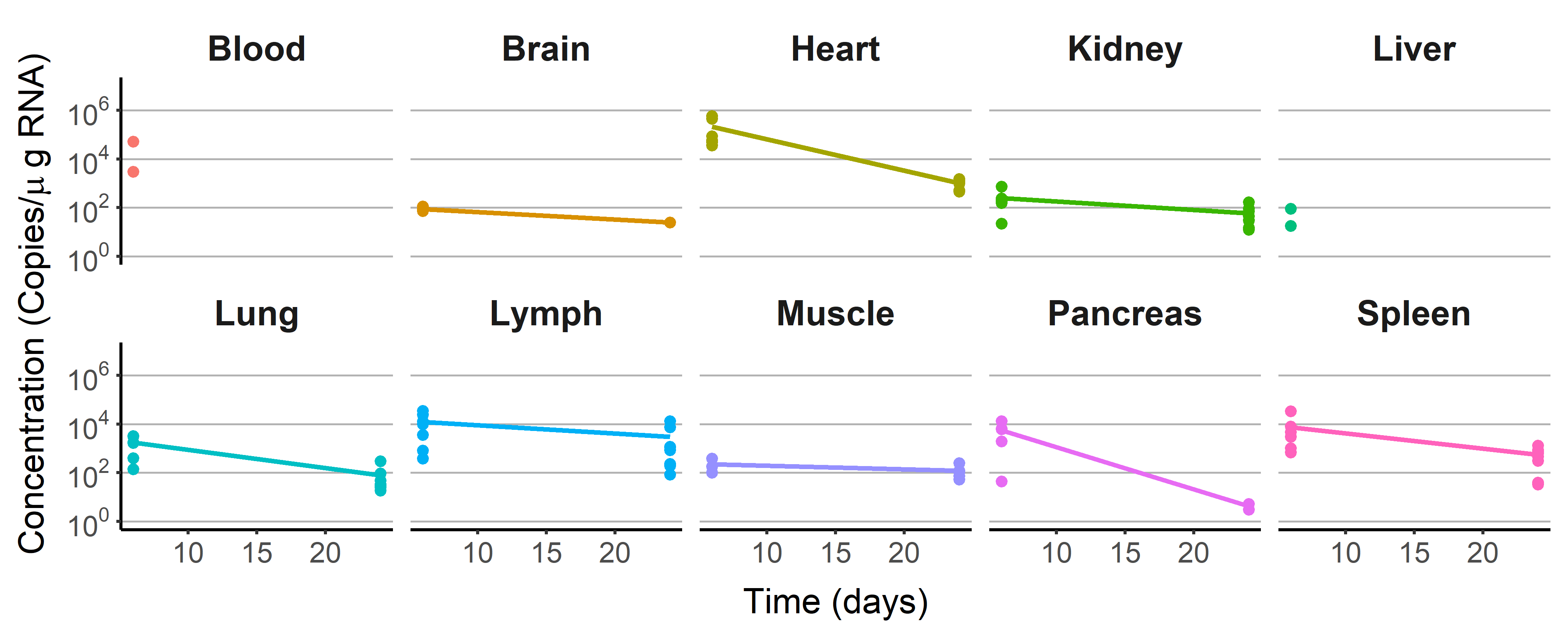


**Supplementary Figure 3**. **SC administration experiment concentration-time profiles.** Dots represent the observations. Solid lines represents median tendency in each organ.


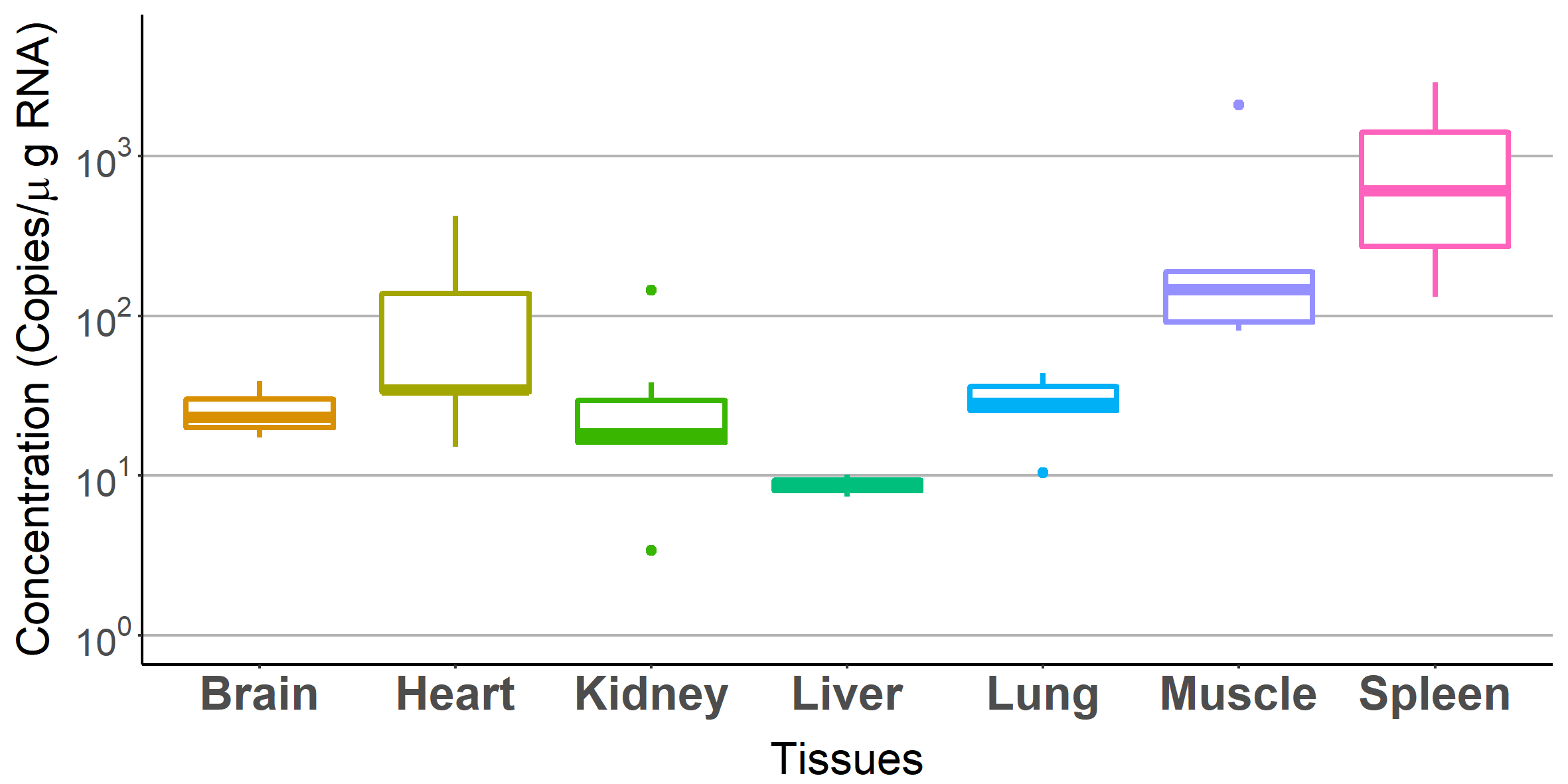


**Supplementary Figure 4. SC administration experiment concentration-time profiles in blood**. Viral concentrations by organs.


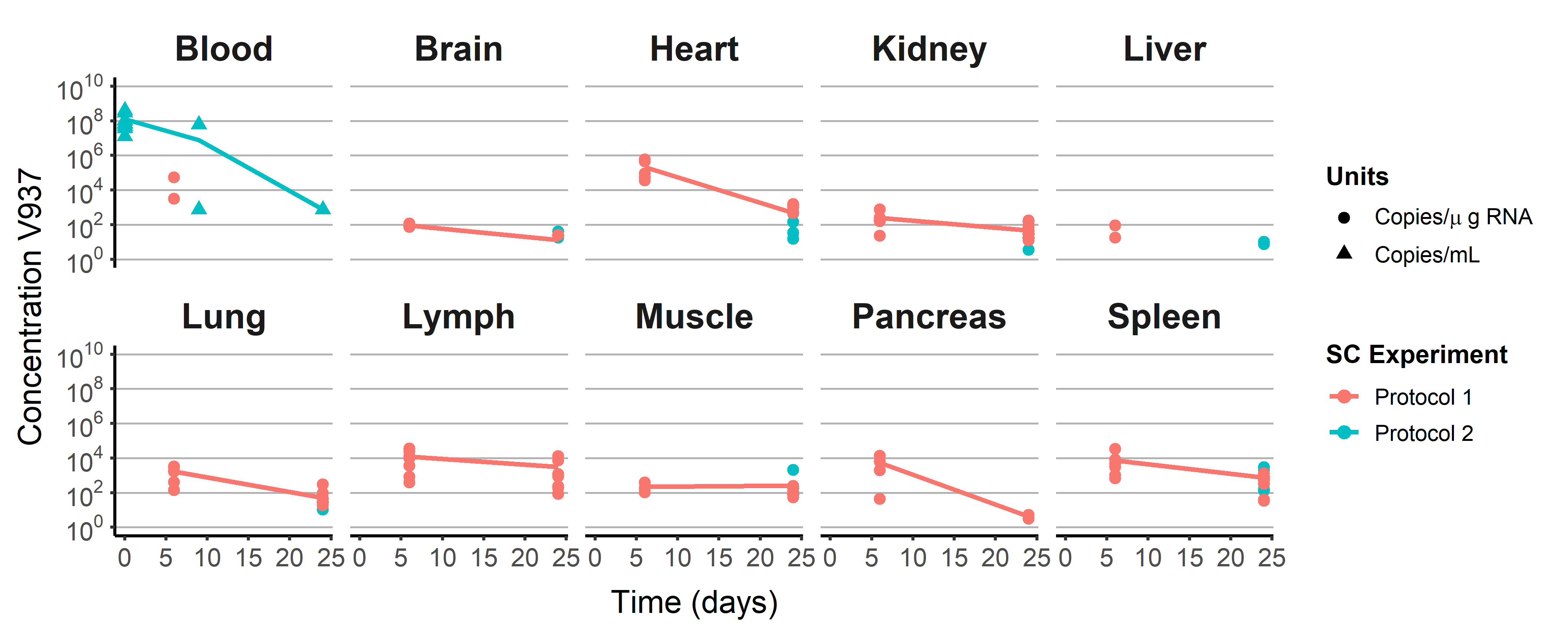


**Supplementary Figure 5.** **Subcutaneous (SC) administration experiments concentration-time profiles.** Dots represent the observations in copies/µg RNA. Triangles represent observations in copies/mL Solid lines represent median tendency in each organ.


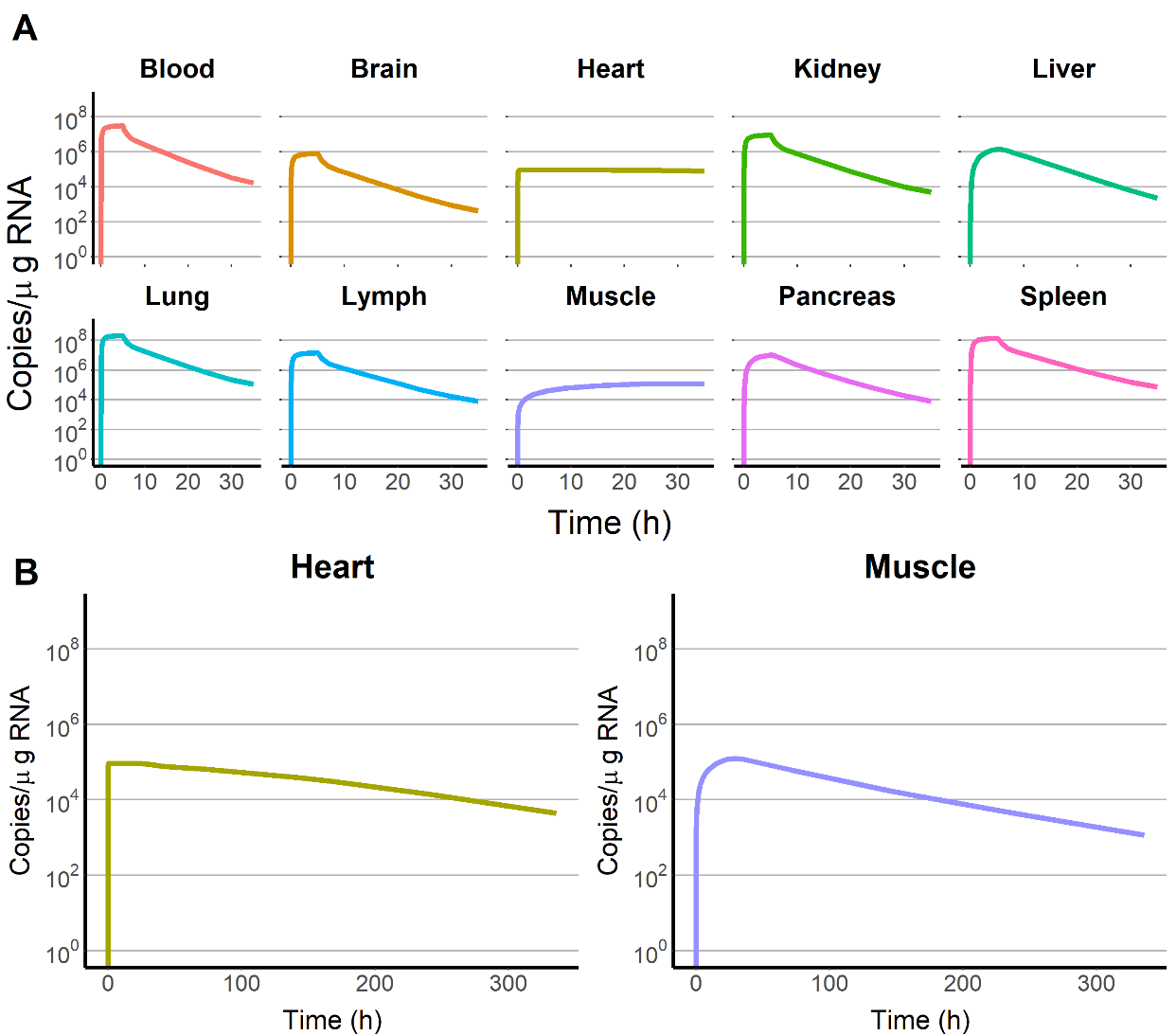


**Supplementary Figure 6** **Final model simulation of 5h intravenous continuous infusion of 2.04 x 10^9^ TCID_50_ of V937.** **A**) Model predicted V937 time course in the different model compartments representing first hours after the infusion time using the final model. **B)** Model predicted V937 time course in heart and muscle tissues demonstrating a delay in elimination.


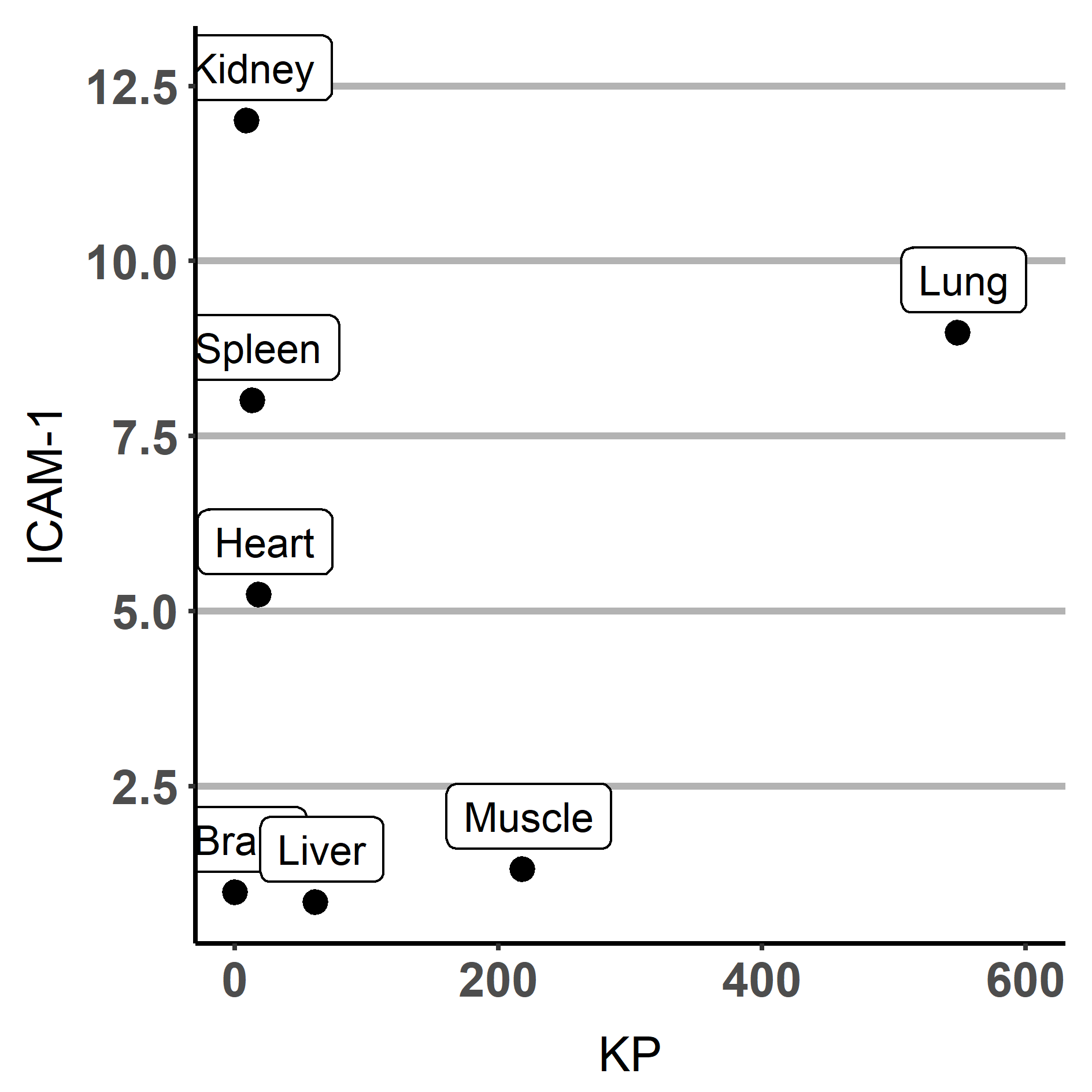


**Supplementary Figure 7.** Scatterplot of the ICAM-1 receptor vs KP parameter.


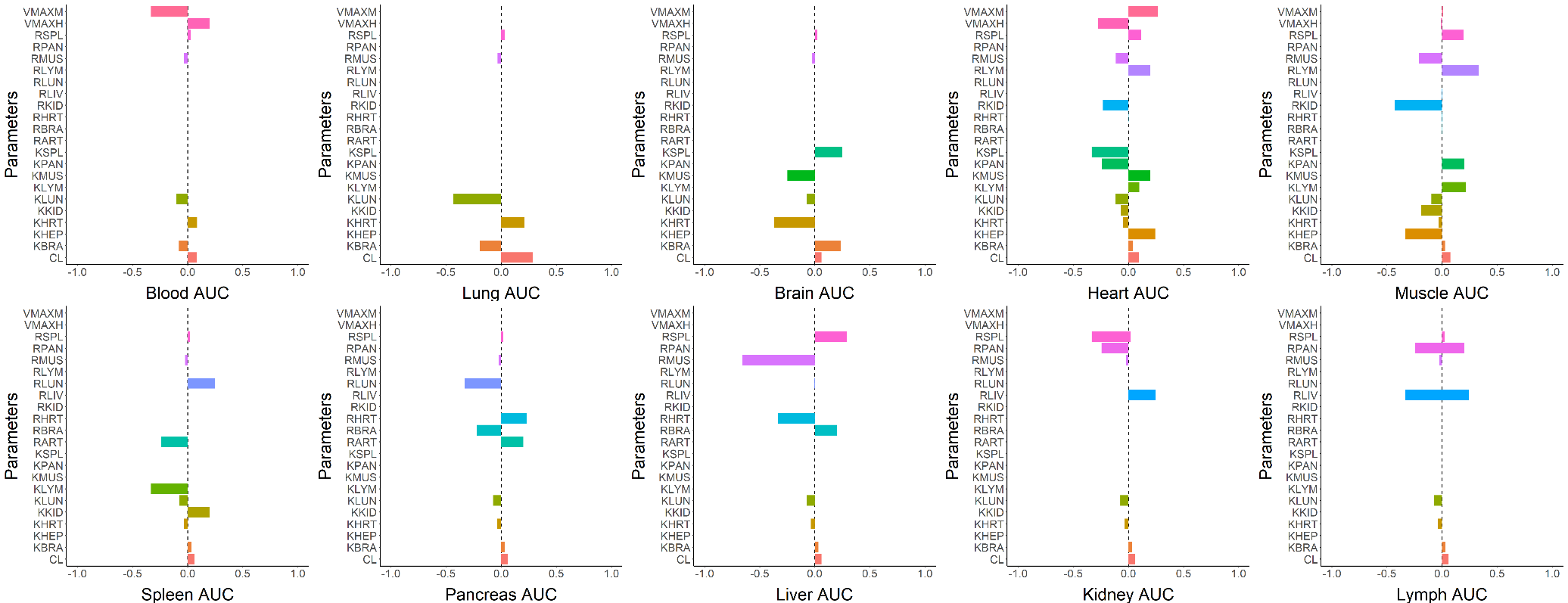


**Supplementary Figure 8.** Sensitivity analysis evaluation of the impact on the AUC_0-tend_ (area under the concentration-time curve from time zero to the last measurable concentration) in different tissues varying model parameters +/- 25% of the point estimates. The x-axis represents the parameter estimates under evaluation, while the y-axis represents the corresponding changes in AUC_0-tend_. Each bar in the graph corresponds to a specific tissue, and the width of the bar indicates the magnitude of change in AUC_0-tend_ resulting from the parameter variation.
