# Supplementary material for: A physiologically based pharmacokinetic model for V937 oncolytic virus in mice
Source: Front Pharmacol. 2023 Sep 13;14:1211452. doi: 10.3389/fphar.2023.1211452 (PMC10524596; doi:10.3389/fphar.2023.1211452)
Supplement: Supplementary file 3 [file DataSheet1.DOCX]

Supplementary Material

A physiologically-based pharmacokinetic model for V937 oncolytic virus in mice

**Sara Peribañez-Dominguez, Zinnia P Parra-Guillen, Tomoko Freshwater, Iñaki F Troconiz**

*** Correspondence:** Zinnia P Parra-Guillen: [zparra@unav.es](mailto:zparra@unav.es)

**APPENDIX**

**Vein blood**

$\frac{{dA}_{ven}}{dt}=input+\sum({Q_{org}\cdot C_{org}}/{{KP}_{org}})-Q_{lun}\cdot\frac{A_{ven}}{V_{ven}}-CL\cdot\frac{A_{ven}}{V_{ven}}$ **(Eq. 1)**

**Arterial blood**

$\frac{{dA}_{art}}{dt}=({Q_{lun}-L_{lun})\cdot C_{lun}}/{{KP}_{lun}}-Q_{art}\cdot\frac{A_{art}}{V_{art}}$ **(Eq. 2)**

**Lung**

$\frac{{dA}_{lun}}{dt}=Q_{lun}\cdot\frac{A_{ven}}{V_{ven}}- ({Q_{lun}-L_{lun})\cdot\frac{A_{lun}}{V_{lun}}}/{{KP}_{lun}}-L_{lun}\cdot\frac{A_{lun}}{V_{lun}}$**(Eq. 3)**

**Liver** $Q_{hep}={Q_{hepa}+Q}_{pan}+Q_{spl}$ **(Eq. 4)**

${\frac{{dA}_{liv}}{dt}=Q}_{hepa}\cdot\frac{A_{art}}{V_{art}}+{\left( Q_{spl}- L_{spl} \right)\cdot\frac{A_{spl}}{V_{spl}}}/{{KP}_{spl}+}{\left( Q_{pan}- L_{pan} \right)\cdot\frac{A_{pan}}{V_{pan}}}/{{KP}_{pan}}-{\left( Q_{hep}- L_{hep} \right)\cdot\frac{A_{hep}}{V_{hep}}}/{{KP}_{hep}}- L_{hep}\cdot C_{hep}$ **(Eq. 5)**

**Spleen**

$\frac{{dA}_{spl}}{dt}=Q_{spl}\cdot\frac{A_{art}}{V_{art}}- {{(Q}_{spl}- L_{spl})\cdot\frac{A_{spl}}{V_{spl}}}/{{KP}_{spl}-}L_{spl}\cdot\frac{A_{spl}}{V_{spl}}$ **(Eq. 6)**

**Pancreas**

$\frac{{dA}_{pan}}{dt}=Q_{pan}\cdot\frac{A_{art}}{V_{art}}- {{(Q}_{pan}- L_{pan})\cdot\frac{A_{pan}}{V_{pan}}}/{{KP}_{pan}-}L_{pan}\cdot\frac{A_{pan}}{V_{pan}}$ **(Eq. 7)**

**Brain**

$\frac{{dA}_{bra}}{dt}=Q_{bra}\cdot\frac{A_{art}}{V_{art}}- {{(Q}_{bra}- L_{bra})\cdot\frac{A_{bra}}{V_{bra}}}/{{KP}_{bra}-}L_{bra}\cdot\frac{A_{bra}}{V_{bra}}$ **(Eq. 8)**

**Heart**

$\frac{{dA}_{hrt}}{dt}=\frac{{VMAX}_{hrt}}{{Km}_{hrt}+\frac{A_{art}}{V_{art}}}\cdot\frac{A_{art}}{V_{art}}- {{(Q}_{hrt}- L_{hrt})\cdot\frac{A_{hrt}}{V_{hrt}}}/{{KP}_{hrt}-}L_{hrt}\cdot\frac{A_{hrt}}{V_{hrt}}$ **(Eq. 9)**

**Muscle**

$\frac{{dA}_{mus}}{dt}=\frac{{VMAX}_{mus}}{{Km}_{mus}+\frac{A_{art}}{V_{art}}}\cdot\frac{A_{art}}{V_{art}}- {{(Q}_{mus}- L_{mus})\cdot\frac{A_{mus}}{V_{mus}}}/{{KP}_{mus}-}L_{mus}\cdot\frac{A_{mus}}{V_{mus}}$ **(Eq. 10)**

**Kidney**

$\frac{{dA}_{kid}}{dt}=Q_{kid}\cdot\frac{A_{art}}{V_{art}}- {{(Q}_{kid}- L_{kid})\cdot\frac{A_{kid}}{V_{kid}}}/{{KP}_{kid}-}L_{kid}\cdot\frac{A_{kid}}{V_{kid}}$ **(Eq. 11)**

**Lymph**

$\frac{{dA}_{lym}}{dt}=L_{lun}\cdot\frac{A_{lun}}{V_{lun}}+L_{hrt}\cdot\frac{A_{hrt}}{V_{hrt}}+L_{mus}\cdot\frac{A_{mus}}{V_{mus}}+L_{spl}\cdot\frac{A_{spl}}{V_{spl}}+L_{pan}\cdot\frac{A_{pan}}{V_{pan}}+L_{hep}\cdot C_{hep}+L_{kid}\cdot\frac{A_{kid}}{V_{kid}}- {Q_{lym}\cdot\frac{A_{lym}}{V_{lym}}}/{{KP}_{lym}}$ **(Eq. 12)**
